# Ameliorative effects of gum Arabic–coated selenium nanoparticles on Cisplatin-induced cerebellar neurotoxicity in rats

**DOI:** 10.1038/s41598-026-37185-8

**Published:** 2026-02-12

**Authors:** Mai Alaa El-Dein, Mohamed A. Marawan, Shaimaa A. Hamouda, Hend Abd El-Halim Mansour, Amoura M. Abou-El Naga

**Affiliations:** 1 Zoology Department, Faculty of Science, Mansoura University, Mansoura, Egypt; 2 Zoology and Entomology Department, Faculty of Science, Al-Azhar University, Nasr City, Cairo Egypt

**Keywords:** Selenium nanoparticles, Arabic gum, Cisplatin, Cerebellum, Antioxidant, Anti-apoptotic agents, Biochemistry, Drug discovery, Neuroscience

## Abstract

Selenium nanoparticles were prepared by using Arabic gum as the stabilizer in a facile synthetic approach. The current study aims to evaluate the possible therapeutic role of alone and combined with on for protecting the cerebellum against cisplatin-induced neurotoxicity. Twenty male albino rats were randomly divided into four groups (5 male/group) as the following: Control group: The rats were injected intraperitoneally with 0.9% saline daily for 30 days. Arabic gum (AG) coated selenium nanoparticles group: The rats received Nano Selenium (0.5 mg/kg) coated with Arab Gum (7.5 mg/kg) orally for 30 days. Cisplatin group: The rats received intraperitoneally a single dose of cisplatin (5 mg/kg) Cisplatin + Arab gum coated selenium nanoparticles group: The rats received intrapritoneally a single dose of cisplatin (5 mg/kg) and nano-selenium (0.5 mg/kg) coated with Arab gum (7.5 mg/kg) orally for 30 days.Combined modality of both cis and AG.SeNPs depicted a significant increased in glutathione (GSH), Catalase (CAT) and Super oxide dismutase (SOD), content. While markedly decreased 8-hydroxy-2’-deoxyguanosine (8-OHdG), Malondialdehyde (MDA) and Hydrogen peroxide (H2O2), levels as compared to cisplatin group. Furthermore, caspase-3 and P53 levels were mitigated. Dopamine and Serotonin levels markedly increased compared to the cisplatin intoxicated group. Moreover, tumor necrosis factor-alpha (TNF-α), cytokine interleukin (IL)-6 (IL-6) and cytokine interleukin (IL)-10 (IL-10) concentrations and level were significantly decreased with treatment. Histological examinations confirmed the recuperating effects of AG.SeNPs on cerebellar tissue intoxicated with cisplatin which was indicated by intact folia, and restored Purkinje cells number and architecture. In conclusion, Selenium nanoparticles coated with Arabic gum could be used as an antioxidant, anti-apoptotic and anti-inflammatory agent to alleviate the toxicity of cisplatin on the rat cerebellum.

## Introduction

The cerebellum develops from the dorsolateral part of the alar laminae of the metencephalon and formed a network of neurons that is highly involved in the adaptive regulation of sensory, motor, and cognitive processes; it is strongly connected to other regions of the brain^1^. The cerebellum is essential for motor control and learning. The relative simplicity of the cerebellar cortical circuit has prompted much research into its functionality, rendering the cerebellum one of the most well-characterized structures in the central nervous system^2^. Cisplatin is a very effective and extensively utilized chemotherapeutic agent for the treatment of various solid tumors^3^. Cisplatin was the inaugural heavy-metal compound utilized as an antineoplastic agent. Furthermore, it has been demonstrated that cisplatin causes the death of cerebellar granule cells in a laboratory setting, leading to both structural and molecular changes during the development of the cerebellum in postnatal rats^4^. Despite significant advancements in oncology, it remains available and continues to be one of the most often utilized medications in cancer treatment^5,6^. One of the negative consequences associated with cisplatin is the occurrence of oxidative stress caused by free radicals, which leads to a decrease in brain antioxidant enzymes^7^.

Numerous medications for hematologic malignancies induce toxicity in both peripheral and central nervous systems (CNS), and treatment-related effects must be prioritized in the differential diagnosis of otherwise unexplained neurological symptoms. Taste receptors and olfactory neurons do regenerate, but they are vulnerable to harm from chemotherapy^8^. These adverse effects lead to a decrease or discontinuation of treatment or significantly affect the quality of life of patients, resulting in elevated levels of negative emotional states such as depression and anxiety. Currently, there is no medicine that effectively prevents these adverse effects. The treatment plan in place focuses on managing symptoms, but its effectiveness is limited^9^.

The application of nanotechnology has been widespread across various domains, with particular emphasis on its utilization in the detection and management of illnesses. Non minerals are minerals that have undergone nanotechnology processing to transform them into minuscule particles^10^. Selenium is an essential micronutrient that is crucial for the overall health of humans, animals, and microorganisms. Selenium nanoparticles are widely used in several biomedical applications due to their high level of bioactivity^11^. Moreover, they have the ability to act as chemo preventive agents, anti-inflammatory agents, and antioxidants^12^. SeNPs were among the numerous nanoparticles that showed antioxidant action^13^. Right now, applying scientific progress, several plants have been studied, and chemicals derived from them have exhibited beneficial therapeutic effects, encompassing anticancer, antibacterial, antioxidant, anti-inflammatory, and immunomodulatory capabilities^14^.

The dried gummy exudate produced by the stems and branches of Acacia senegal or Acacia seyal trees is turned into gum Arabic, which is also called gum Acacia. It is a dietary fibrous heteropolysaccharide that is edible and soluble in water. Agroforestry systems cultivate gum Arabic as a cash crop^15^. Arabic gum is utilized in medications, where it has various therapeutic benefits due to its antibacterial, anti-inflammatory, and antioxidant effects^16^. Some studies conducted in recent years have shown that Arabic gum can mitigate or even eliminate the harmful side effects of some commonly used medications, including chemotherapy and analgesic. Coating selenium nanoparticles with gum Arabic is a simple procedure that improves their stability and biocompatibility. This coating technique produces selenium nanoparticles exhibiting superior colloidal stability, higher dispensability in aqueous environments, and augmented biocompatibility^17^.

This study aimed to investigate the therapeutic effectiveness of coating selenium nanoparticles with gum Arabic as an anti-tumor plant. Additionally, it examined the antioxidant and IL-6, IL-10 inflammatory markers, that influence the regulation of central nervous system health and neural development in cisplatin-induced cerebellar damage in adult male albino rats.

## Materials and methods

### Ethics and consent to participate declarations

All experimental procedures complied with established institutional, national, and international regulations governing the care and use of laboratory animals. Measures were implemented to minimize animal discomfort and to limit animal numbers while maintaining the scientific rigor and reproducibility of the study. All protocols modified for this study have been in compliance with Mansoura University’s Institutional Animal Ethics Committee’s clearance. The Ethics Committee of the Laboratory Animals at Mansoura University’s Faculty of Science gave its approval to this work (Code No: MU-ACUC (SC.R.24.11.19)). The study has been prepared and reported in full accordance with the ARRIVE guidelines for animal research as stated in their article (NIH article No. 85 − 23, amended 1996).

### Chemicals

Arabic gum CAS#: 9000-01-5, Lot Number: 822,140. Nano selenium, and cisplatin EIMC Pharmaceuticals CO, Cairo, Egypt CAS#: 15663-27-1. All other reagents were of high analytical quality and acquired from Sigma-Aldrich Chemical Company Egypt.

### Phytochemical analysis of Arabic gum (HPLC)

An Agilent Series 1100 HPLC apparatus (Agilent, USA) comprising an auto-sampling injector, solvent degasser, two LC pumps (series 1100), ChemStation software, and a UV/Vis detector set at 250 nm for phenolic acids and 360 nm for flavonoids was used to analyse phenolic and flavonoid compounds. A C18 column (125 mm × 4.60 mm, 5 μm particle size) was used for the study. A gradient mobile phase comprising two solvents, Solvent A (methanol) and Solvent B (acetic acid in water at a 1:25 ratio), was used to separate phenolic acids. For the first three minutes of the gradient program, the concentration remained at 100% B. The process involved applying 50% eluent A for five minutes, rising to 80% for two minutes, and then lowering to 50% for five more minutes. The detection took place at a wavelength of 250 nm. A mobile phase comprising acetonitrile (A) and 0.2% (v/v) aqueous formic acid (B) with an isocratic elution program of 70:30 was used to isolate flavonoids. The separation took place at 25 °C with a solvent flow rate of 1 ml/min. 25 µL was the injection volume.

### Preparation of liquid AG

The AG powder was purchased from a local supermarket, extracted from the Sudanese tree Acacia Senegal, the liquid AG was prepared by dissolving it in heating water^18^.

### Preparation of selenium nanoparticles

The hydrothermal method for Synthesizing selenium nanoparticles using thyme extract at 60 °C for 24 h is an ecologically friendly approach. The procedure typically involves mixing a selenium precursor (sodium selenite) with aqueous thyme extract inside a sealed autoclave or hydrothermal reactor. The mixture is then heated to 60 °C and maintained at this temperature for 24 h, allowing the bioactive compounds in the thyme extract to decrease selenium ions and facilitate the formation of nanoparticles^19^. The relatively mild temperature and extended reaction time promote controlled, slow development of the nanoparticles, possibly resulting in a narrow size distribution. The phytogenic capping agents from thyme extract prevent agglomeration and stabilize the produced nanoparticles. Following the reaction time, the fluid is cooled, and the selenium nanoparticles are separated, purified, and dried for further characterization and application. This method employs the antioxidant properties of thyme to generate biocompatible selenium nanoparticles, potentially applicable in biomedicine and agriculture^20^.

### Preparation of Arab gum-coated selenium nanoparticles composite

Selenium nanoparticles coated with Arabic gum by the hydrothermal method. Coating selenium nanoparticles with gum Arabic is a straightforward process that enhances their durability and biocompatibility. Subsequent to the manufacture of selenium nanoparticles using the hydrothermal method, they are dispersed in an aqueous solution and mixed with a prepared Arabic gum solution. The mixture is then stirred, often at room temperature or with mild heating (50 °C), allowing the Arabic gum to form a protective layer around the nanoparticles. The coated particles are then extracted using centrifugation or filtration, purified to remove excess Arabic gum, and dehydrated, sometimes utilizing freeze-drying methods. This coating method generates selenium nanoparticles that demonstrate enhanced colloidal stability, increased dispersibility in aqueous mediums, and improved biocompatibility. The Arabic gum coating protects the nanoparticles from oxidation and improves their use in targeted drug delivery systems. The properties of the coating may be enhanced by adjusting factors such as Arabic gum content, reaction time, and temperature during the process^21^.

### Zeta analysis and TEM for selenium nanoparticles and selenium nanoparticles coated with Arabic gum

**Fig. 1:**
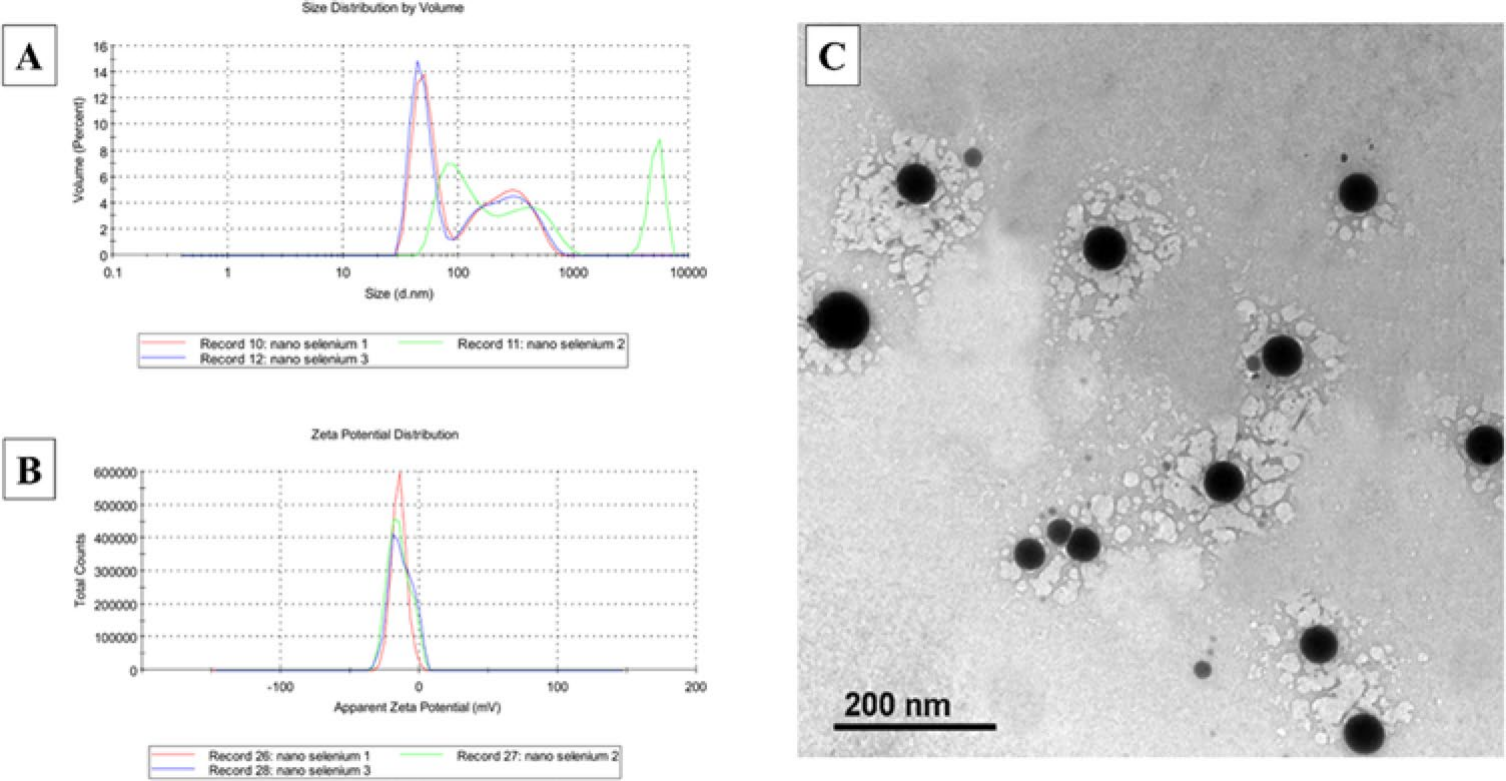
Zeta analysis and transmission electron microscopy (TEM) for selenium nanoparticles. (**C**) Characterization of selenium nanoparticles (SeNPs). The sample particle size was around 60 to 80 nm. Without aggregation in particles.

The zeta potential and particle size of the synthesized nanoparticles were analyzed using dynamic light scattering (DLS) with a Zeta-sizer (Nano ZS, Malvern Instruments Ltd., Malvern, UK). The synthesized nanoparticles were suitably diluted prior to measurements. The samples were then transferred to a 4 ml quartz cuvette and tested at an ambient temperature (25 °C). Size distributions were analyzed regarding intensity in relation to particle size as shown in Fig. 1.

**Fig. 2:**
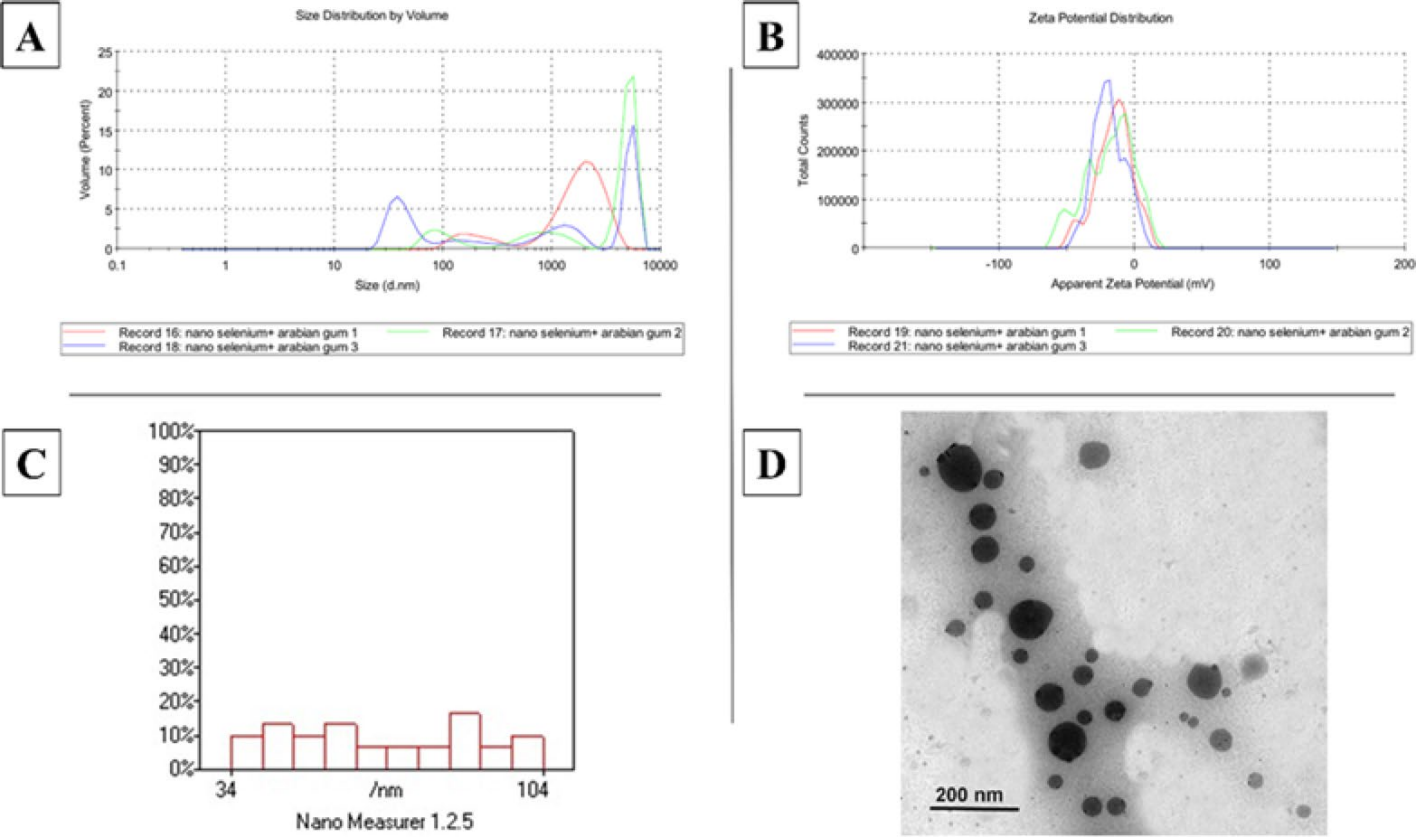
Zeta analysis and transmission electron microscopy (TEM) analysis of the selenium nanoparticles coated with Arabic gum. (**A**) zeta size for selenium nanoparticles coated with Arabic gum. (**B**) Zeta potential for selenium nanoparticles coated with Arabic gum. (**C**) size distributions histogram of prepared SeNPs coated with Arabic gum. (**D**) Characterization of Selenium Nanoparticles SeNPs coated with Arabic gum. The sample of SeNPs-AG possesses variation in size and shape of the spheres with a particle size of around 34–100 nm. There is no evidence of agglomeration, and the particles are Mult dispersed without colonies.

In the current study, Fig. 2, showing selenium nanoparticles (SeNPs) were successfully coated with gum Arabic to enhance their stability and biocompatibility. revealed a bimodal size distribution of SeNPs with a Z-average size of 211.5 nm and a polydispersity index of 0.213. Two distinct peaks were observed at 276.3 nm and 51.91 nm, contributing 46.1% and 53.9% of the total volume, respectively. The ζ-potential of −13.2 mV suggests good colloidal stability, as the high absolute value of the ζ-potential promotes electrostatic repulsion between particles, preventing aggregation. Zeta analysis revealed a polydisperse population of gum Arabic-coated SeNPs with a Z-average diameter of 295.2 nm and a high polydispersity index of 0.853. The presence of multiple peaks, including a significant peak around 1213 nm, suggests a heterogeneous population with a considerable fraction of larger particles. The ζ-potential of −18.8 mV for gum Arabic-coated SeNPs indicates good colloidal stability, as the high absolute value of the ζ-potential promotes electrostatic repulsion, preventing aggregation. The ζ-potential represents the potential difference at the sliding surface of a particle when subjected to an electric field. It measures the potential difference between the electric double layer (EDL) of electrophoretic mobile particles and the surrounding dispersion medium (aqueous or organic) at the sliding plane^22,23^.

### Experimental animals

Rats utilized in this study were acquired from the Egyptian Vaccine Company (VACSERA, Giza, Egypt), which was located in the experimental animal center of the Faculty of Science at Mansoura University. Twenty mature male Sprague Dawley rats, weighing 200 ± 20 gm and aged 8–10 weeks, will be kept in polycarbonate cages, with five rats per cage. They will be kept in a regulated setting with a 12-hour light-dark cycle, air conditioning set at 24 degrees Celsius, and humidity levels between 50 and 70%. There will be unlimited access to food and water during the experiment.

### Experimental design

The male rats were divided randomly into four groups, each group contain 6 rats:

- Control group: The rats injected intraperitoneally (i.p.) with 0.9% saline daily.
- AG. SeNPs. group: The rats received Nano Selenium (0.5 mg/kg)^23^coated with Arab Gum (7.5 mg/kg) orally^24^.
- Cis group: The rats received a single i.p. dose of Cisplatin (5 mg/kg)^25^.
- Cis + AG. SeNPs. group: The rats received single i.p. dose of Cisplatin (5 mg/kg) then Nano selenium (0.5 mg/kg) coated with Arab Gum (7.5 mg/kg) orally.

At the end of the experiment, all male rats in each group were weighed after 30 days, fasted for a full night, and received intraperitoneal injections of 75 mg/kg ketamine26 and 6 mg/kg xylazine^26^. After that, all of the animals were killed by cervical dislocation. An attempt was made to reduce the suffering of the animals. This involved handling animals with care, providing suitable environmental enrichment in their home, and routinely checking for indications of discomfort or pain. When necessary, interventions were implemented right away to reduce discomfort. This approach was selected to guarantee a speedy and painless process.

### Isolation of cerebellum and cerebellar homogenate preparation

Male rats were promptly dissected, and their cerebella were immediately removed. Each cerebellum was rinsed with cold 0.9% saline, cleaned, and dried using lint-free tissue. A 0.3 g portion of the left cerebellum was homogenized, and then centrifuged at 12,000 ×g for 20 min at 4 °C. The resulting supernatant was collected for biochemical analysis^27^.

### Biomarkers analyses

Blood samples were collected for biochemical examination, coagulated at room temperature, and centrifuged for 10 min at 3000 rpm to create clear aliquots. These were then stored in many Eppendorf tubes at −20 °C for further biochemical tests in serum. The antioxidant markers level of GSH, CAT, SOD, 8-OHDG Catalog Number CSB-E10526r, Cusabio Technology, LLC, USA), MDA and H2O2 Sigma-Aldrich, catalog number: 216763) levels will be determined in cerebellar tissues using commercial colorimetric kit (Bio-Diagnostics, Giza, Egypt). The levels of Caspase-3 (Catalog # MBS733100, MBS451593) were quantified using specific immunoassays and p53 (Catalog # MBS721665) were obtained from My BioSource (San Diego, CA, USA). Protein extracts from tissue samples were prepared, and the assays were conducted following the protocols provided by the kit manufacturers to ensure accuracy and reliability of the results^28^. The levels of neurotransmitters as serotonin levels were performed using a kit designated by the code E-El-0033. Eagle Biosciences’ Mouse/Rat Dopamine ELISA Assay Kit (20 A Northwest Blvd., Suite 112, Nashua, NH 03063, USA), Catalogue #: DOU39-K01, was used to measure the amount of dopamine in serum. According to the guidelines provided by the kit makers, TNF-α and IL-10 and IL-6 were quantified by the ELISA method using commercial ELISA kits^29^.

### Histopathological studies and microscopic investigation

Neutral buffered formalin solution (10%) was used to carefully fix the samples of cerebellar tissue. The dehydration process was completed in increasing ethyl alcohol grades, followed by xylene clearing and embedding in extremely pure paraffin wax. Hematoxylin and eosin stain 30 was used to stain the 5–7 μm slices that had been prepared and soaked in a decreasing series of ethanol. In order to identify histopathological changes, the stained sections were further inspected and captured on camera using an Olympus light microscope (Amscope MU1000). A Purkinje cell count was then conducted using five distinct fields/ml per sample, followed by statistical analysis^30^. A semi-quantitative histological scoring system was utilized to assess the extent of neurodegeneration. Five distinct fields from each section (*n* = 5 per group) were analyzed for edema, cytoplasmic vacuolation, and nuclear pyknosis. Each parameter was evaluated on a scale from 0 to 4 according to the severity of the lesion: 0 (normal), 1 (< 10%), 2 (10–25%), 3 (26–50%), and 4 (> 50%). The final ‎score for each animal was the mean of the assessed fields, and the data were analyzed using Kruskal-Wallis test followed by Dunn’s post-hoc test for multiple comparisons.

### Immuno-histochemical (IHC) investigations

Paraffin-embedded cerebellar sections were deparaffinized in xylene and processed for immunohistochemical (IHC) staining using the labeled streptavidin–biotin immunoperoxidase method, following established protocols. Antigen retrieval was performed by immersing the sections in 10 mM citrate buffer (pH 6.0), heating them to boiling in a Gibson microwave oven (USA) at full power, and then allowing them to cool at room temperature for 10–20 min. This microwave-based technique enhances antigen exposure. Endogenous peroxidase activity was quenched using 3% hydrogen peroxide (H₂O₂) in phosphate-buffered saline (PBS; 10 mM sodium phosphate, 140 mM sodium chloride, pH 7.2). The sections were then washed three times in PBS for two minutes each. To prevent non-specific antibody binding, a blocking serum was applied for 10 min. Excess blocking serum was removed before incubating the sections with primary antibodies targeting GFAP and NFL. Immunostaining was carried out using the Power-Stain™ 1.0 Poly HRP AEC Kit (Genemed Biotechnologies, Inc., Cat. #54 − 0022) with diaminobenzidine (DAB) as the chromogen. Appropriate positive controls were included to validate the staining protocol^30^.

### Ultrastructural examination

Cerebellar tissues were promptly isolated and fixed in 4% glutaraldehyde prepared in Dulbecco’s modified phosphate-buffered saline. The samples were then post-fixed in 1% osmium tetroxide for one hour at room temperature, followed by thorough washing. After fixation, the tissues underwent dehydration through a graded ethanol series, treatment with propylene oxide, and embedding in Epon 812 resin (Fluka Chemie, Switzerland). Ultrathin Sects. (60–70 nm) were prepared using a diamond knife on an LKB ultramicrotome. These sections were mounted on copper grids, stained with uranyl acetate and lead citrate, and examined using a JEOL 2100 transmission electron microscope (TEM) operating at 80 kV. The analysis was conducted at the Faculty of Agriculture, Mansoura University, Egypt^31^.

### Molecular docking assay

This assay was performed to estimate the direct interaction between some compound extracted from Arabic gum, identified through HPLC analysis, include flavonoids and phenolic acids with Markers associated with anti-apoptotic and anti-inflammatory activities such as Caspase-3, TNF-α and IL-6. The chemical structures were obtained from the Drug Bank^32,33^. The production of ligands was conducted using the SMILES format, then imported into Avogadro software to construct 3D Structures and perform energy minimization. The protein structure was transformed from PDB to PDBQT format by open babel GUI software and both the protein and ligand were imported into PYRX for docking simulations. Several software tools, such as PYMOL, chimera X and Discovery studio2024^34^, were used for visualization. SwissADME: a free web tool to evaluate pharmacokinetics, drug-likeness and medicinal chemistry friendliness of compound extracted from Arabic gum^35^.

### Statistical analysis

One-way ANOVA test was used to statistically analyse the current data for each experimental group in accordance with the mathematical description given by the statistical software application Prism (GraphPad Prism, 6.01). The results are displayed as the five samples’ mean ± standard error of the mean (SEM). A significant difference was deemed to exist when the P value was less than 0.05.

## Results

### AG phytochemical analysis

HPLC analysis results presented in Table 1 displayed antioxidant contents in the Arabic gum, (a) Flavonoids compounds are 4.3,7,8,9 and 12 peaks of the retention time (RT). The RT peaks for (b) Phenolic compounds are 5, 6,7,8,10 and 11.

**Table 1:** HPLC chromatograms of (a) flavonoids of AG. HPLC chromatograms of (b) of phenolic compounds.


RT#	Compound		Concentration (µg/ml)		Biological activity	Reference
4.3	Naringin		3.36		Anti-inflammatory and antioxidant properties Anticancer activity Pharmacokinetics and metabolism	^36^ ^37^ ^38^
7.0	Quercetin		8.76		Neuroprotective effects Anti-inflammatory and antioxidant properties	^39^
8.0	Kaempferol		6.74		Therapeutic potential Anti-cancer roles and mechanisms	^40^ ^41^
9.0	Luteolin		14.69		Antioxidant and anti-inflammatory properties Neuroprotective effects	^42^
12.0	Catechin		3.52		antioxidant properties Anti-inflammatory and anti-allergenic effects	^43^


RT#	Compound		Concentration (µg/ml)		Biological activity	Reference
5.0	Syringic		6.72		Antibacterial activity	^44^
6.0	P-Coumaric		14.70		Pharmacological potentials Therapeutic perspectives	^45^ ^46^
7.0	Cinnamic		0.88		Antioxidant properties Neuroprotective effects	^47^
8.0	Caffeic		13.86		Antioxidant properties Neuroprotective effects	^48^ ^49^
10.0	Gallic		5.45		Anti-inflammatory effects Antioxidant properties	^50^
11.0	Ferulic		14.97		Antimicrobial and antiviral Activity Anti-inflammatory effects	^51^ ^52^

### Impact of AG. SeNPs. on neurotransmitters

**Fig. 3:**
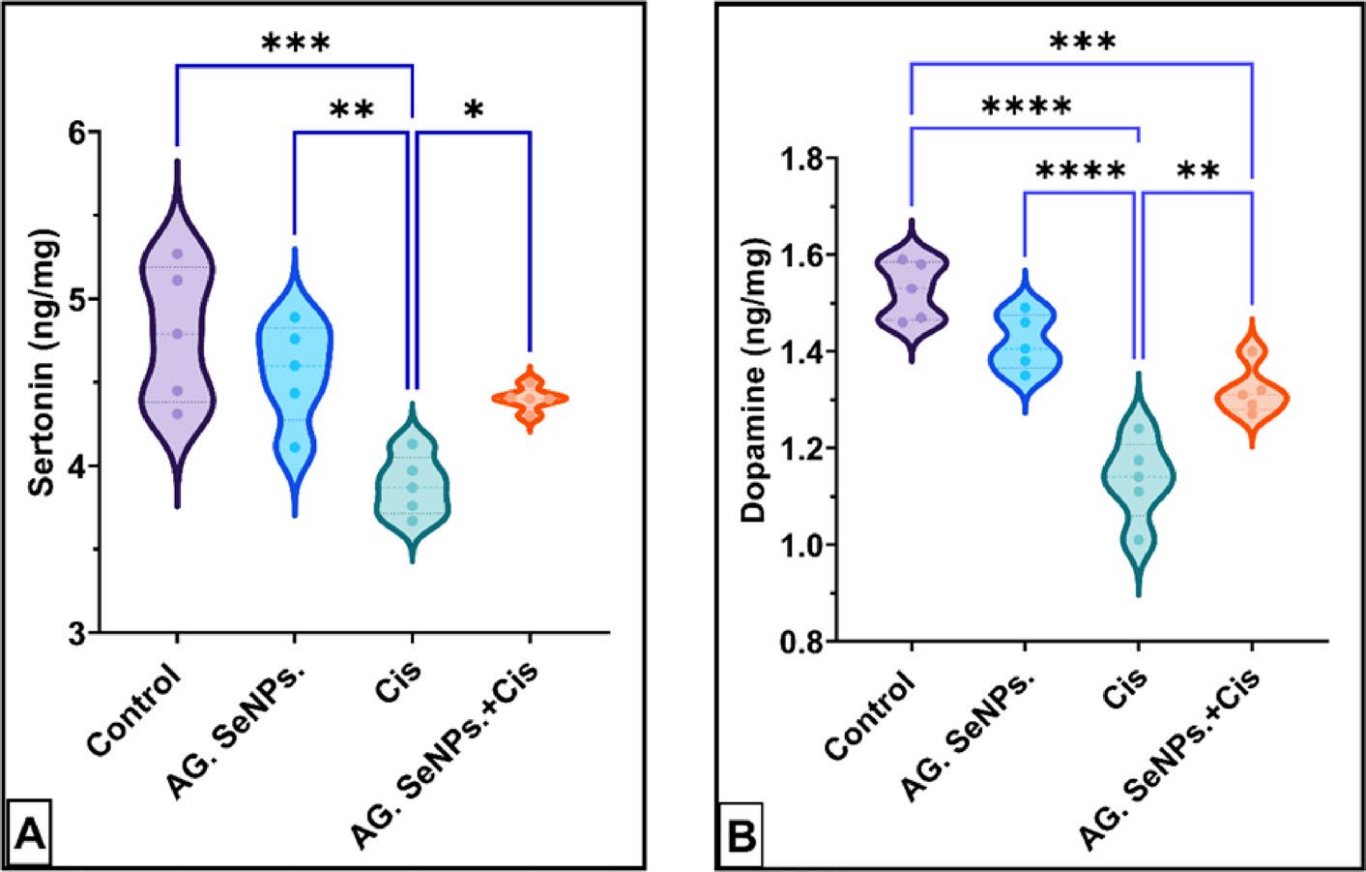
Effect of AG. SeNPs. on cerebellar: (**A**) sertonin (ng/mg); (**B**) dopamine (ng/mg). Values are expressed as means ± SEM; (n=6), * (P<0.05), ** (P<0.01), ***(P<00.001) and ****(P<0.0001).

Cisplatin cerebellar toxicity was clearly demonstrated via the significant depletion of neurotransmitter levels serotonin and dopamine (18.93% and 25.6225%, P<0.0001) respectively compared to the control group. Treated group with AG. SeNPs. markedly improved this depletion in both serotonin and dopamine levels (17.5%, P<0.01) and 24.84%, P<0.0001) respectively compared to the Cis group as shown in Fig. 3A,B.

### Impact of AG. SeNPs. On the oxidative stress markers

**Fig. 4:**
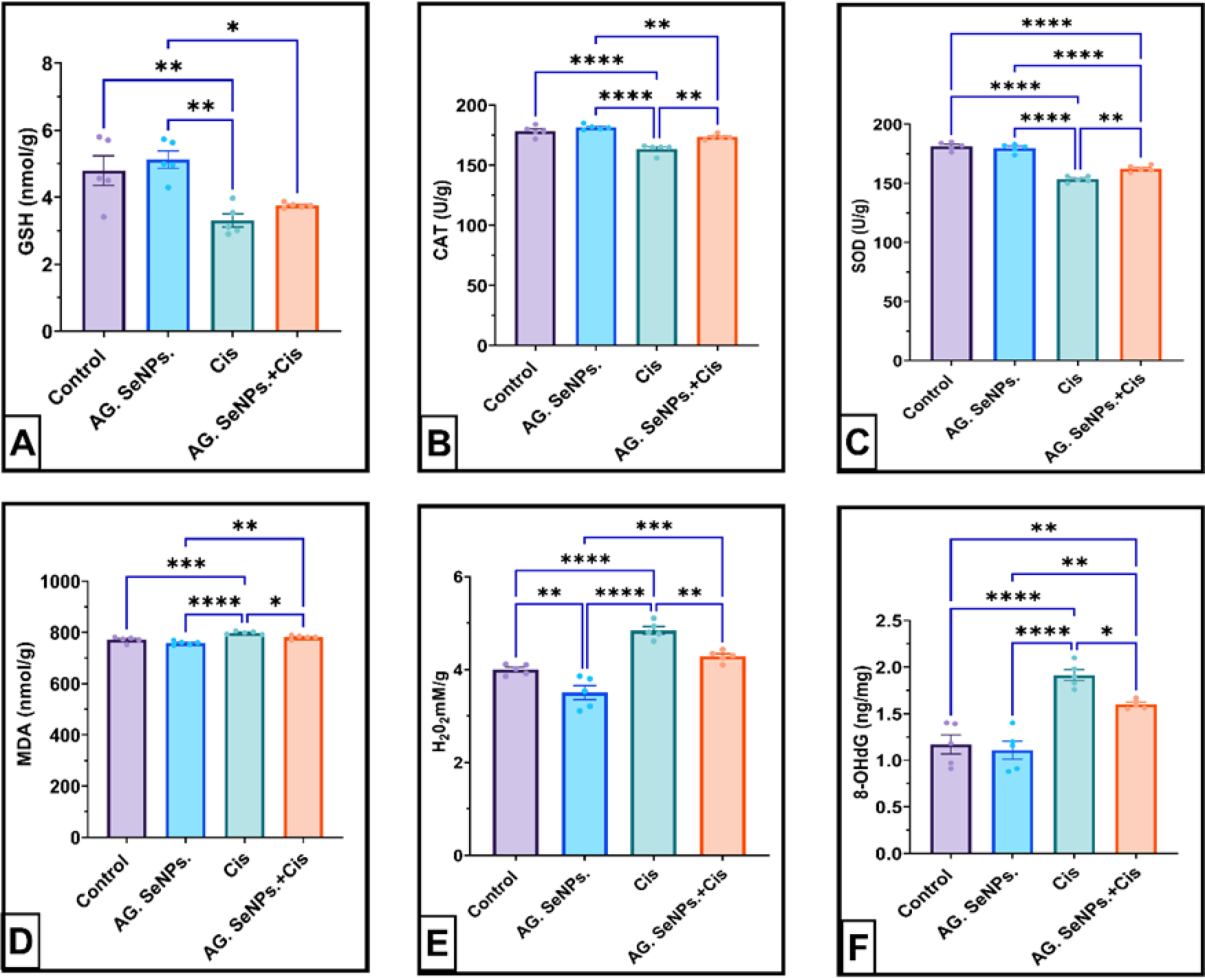
(**A**–**F**): Effect of AG. SeNPs. On: (**A**) serum GSH nm/g; (**B**) serum CAT (U/g); (**C**) serum SOD (U/g); (**D**) serum MDA nm/g; (**E**) serum H_2_O_2_ mM/g; (**F**) serum 8-OHDG ng/mg in the control and treated groups. Values are expressed as means ± SEM; (*n* = 6), *(*P* > 0.05), ** (*P* > 0.01), ***(*P* > 0.001) and ****(*P* > 0.0001).

Comparing the Arabic gum-coated selenium nanoparticles to the control group revealed no discernible changes. When compared to the negative control group, the cisplatin group’s antioxidant levels, GSH, CAT, and SOD, were considerably lower (*P* < 0.0001) after receiving cisplatin (31.0244, 5.37, and 15.4914%). Meantime, Cis + AG. SeNPs. group markedly improved serum GSH, CAT, and SOD levels (54.869, 7.559, and 17.1968, *P* > 0.01%) respectively compared to the cisplatin group as shown in Fig. 4A–C. Cisplatin group have a significant change in level 8-hydroxy-2’-deoxyguanosine (8-OHDG), Malondialdehyde (MDA) and Hydrogen peroxide (H_2_O_2_) was significantly increased as biomarkers for lipid peroxidation and DNA oxidation after administering cis platin (*P* > 0.0001 For 8-OHDG and H_2_O_2_, but *P* > 0.001 For MDA) (63.589, 2.99447, and 21.14%) respectively compared to the negative control group. Treated group with selenium nanoparticles coated with Arabic gum markedly reduced 8-hydroxy-2’-deoxyguanosine (8-OHDG), Malondialdehyde (MDA) and Hydrogen peroxide (H_2_O_2_) levels (42.0585, 27.653, and 4.438%, *P* < 0.0001) respectively compared to the cisplatin group as shown in Fig. 4D–F.

### Inflammatory and apoptotic markers

**Fig. 5:**
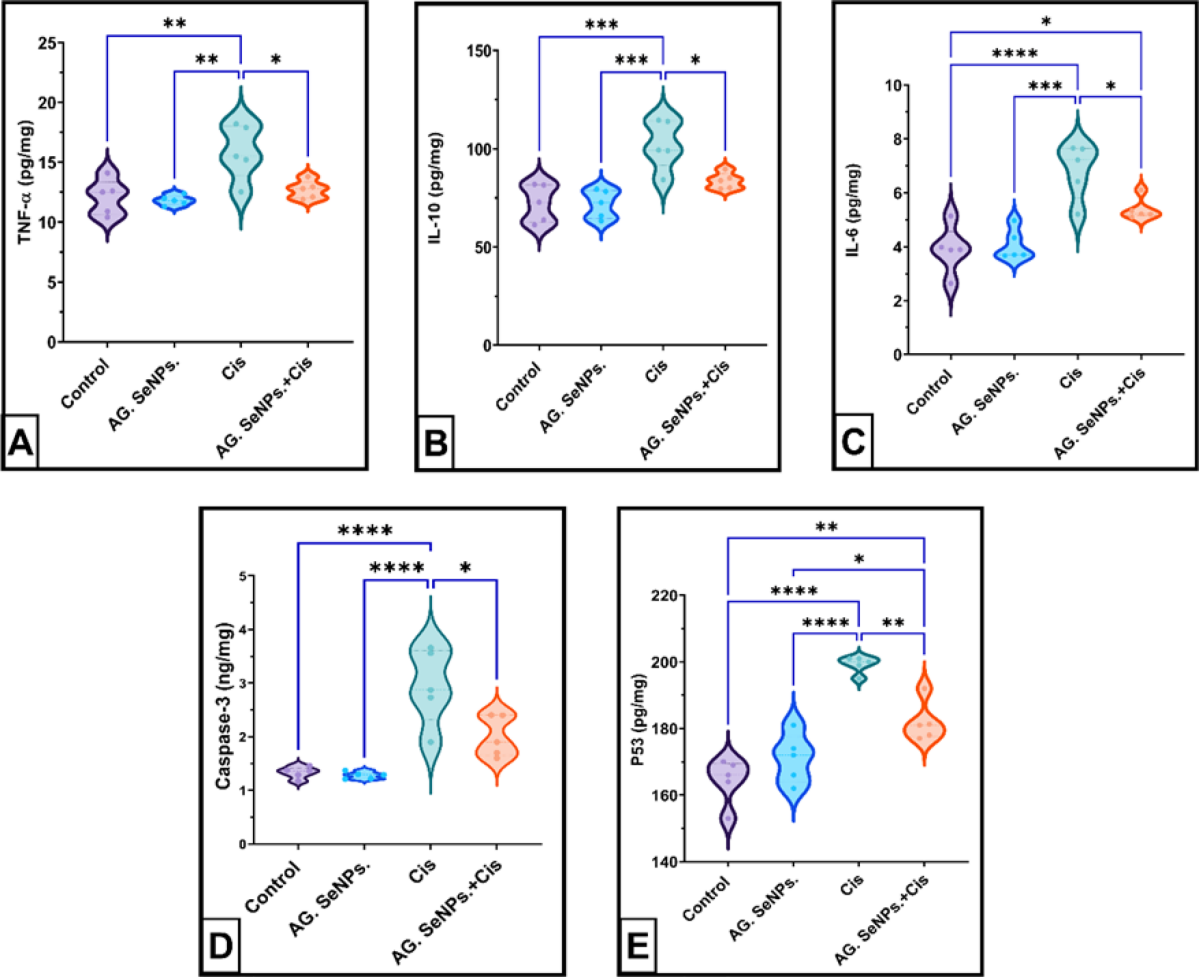
(**A**–**D**): Effect of AG. SeNPs on cerebellar: (**A**) TNF- (pg/mg); (**B**) IL-10 (pg/mg); (**C**) IL-6 (pg/mg) (**D**) caspase-3 (ng/mg); (**E**) P53(pg/mg)in the control and treated groups. Values are expressed as means ± SEM; (n = 6), ‎*(P‏<0.05), ** (P<0.01), ***(P<0.001) and ****(P<‏‎0.0001).

Cisplatin intoxication resulted in a remarkable elevation in the cerebellar tissue inflammatory cytokines levels (31.116%, P< 0.01 for TNF-), (41.46%, P<0.001 for IL-10) and 74.8%, P< 0.0001 for IL-6) respectively compared to the untreated control group. While cotreatment with AG. SeNPs. markedly alleviated these abnormalities for TNF- , IL-10 and IL-6 levels (P<0.01, *P* < 0.001 and *P* < 0.05) (25.51, 29.65 and 40.31%) respectively compared to the cisplatin group; this data represented in Fig. 5A–C.

As depicted in Fig. 5D,E, the Cisplatin group exhibited a significant alteration in apoptotic markers, with caspase-3 and P53 levels markedly elevated following cisplatin administration (P<‏>‏0.0001), showing increases of 121.05% and 21.167%, respectively, in comparison to the negative control group. The treated group receiving selenium nanoparticles coated with Arabic gum exhibited a significant reduction in cerebellar caspase-3 and P53 levels (P<0.0001) of 56.58% and 14.16%, respectively, in comparison to the cisplatin group.

### Histopathological examination

**Fig. 6:**
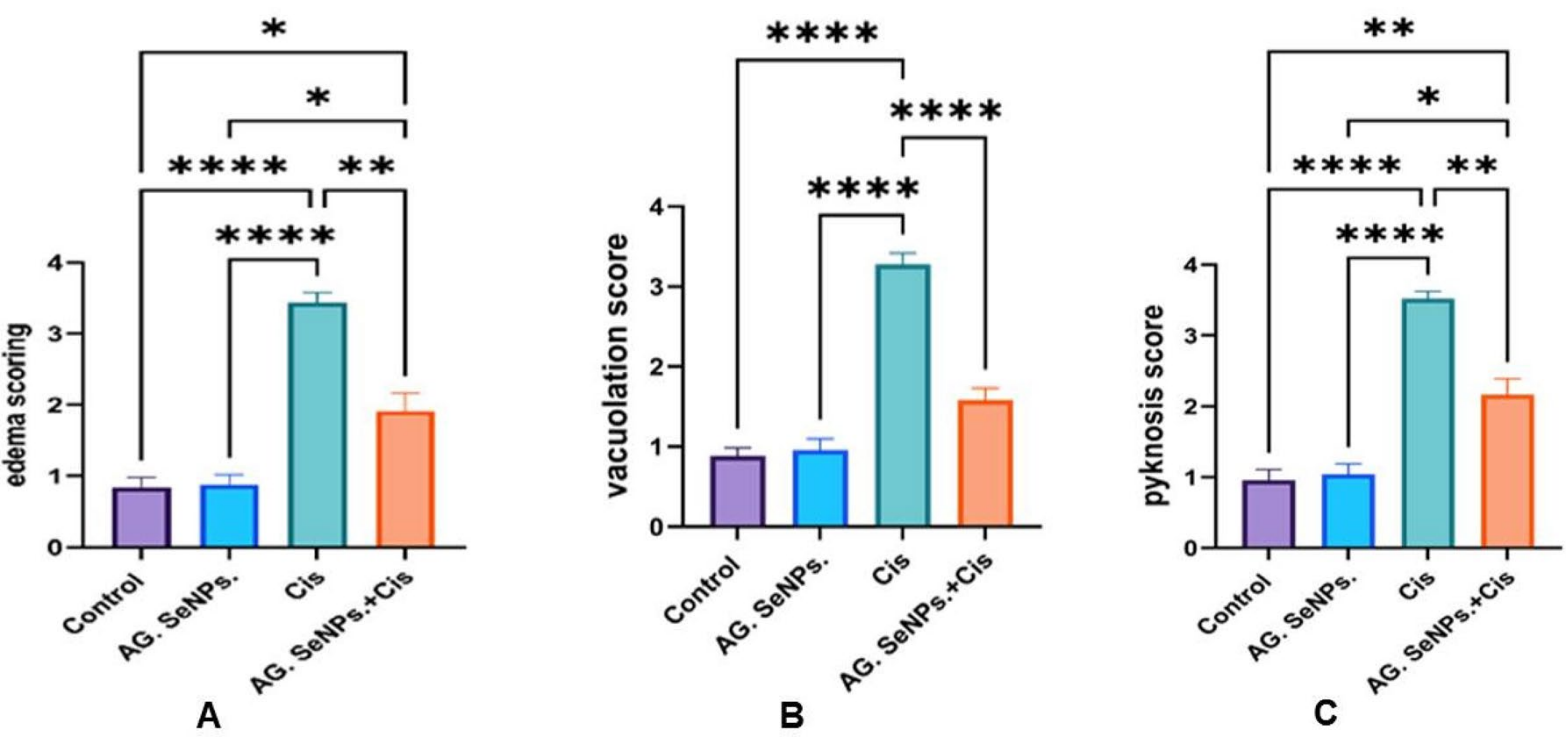
(**A**–**C**): Effect of AG. SeNPs on cerebellar: (**A**) edema scoring; (**B**) vacuolation score; (**C**) pyknosis score in the control and treated groups. Values are expressed as means ± SEM; (n = 6), *(P<0.05), ** (P<0.01), ***(P<0.001) and ****(P<0.0001).

The histopathological score for the control and AG-SeNPs ‎ group indicated normal architecture, while Cis group depicted high pathological lesion score (3–4) indicating sever toxicity. Cotreatment revealed a marked depletion in edema, vacuolation and pyknosis score confirming the neuroprotective efficacy of the nanoparticles through objective quantification (Table 2; Fig. 6).

**Fig. 7:**
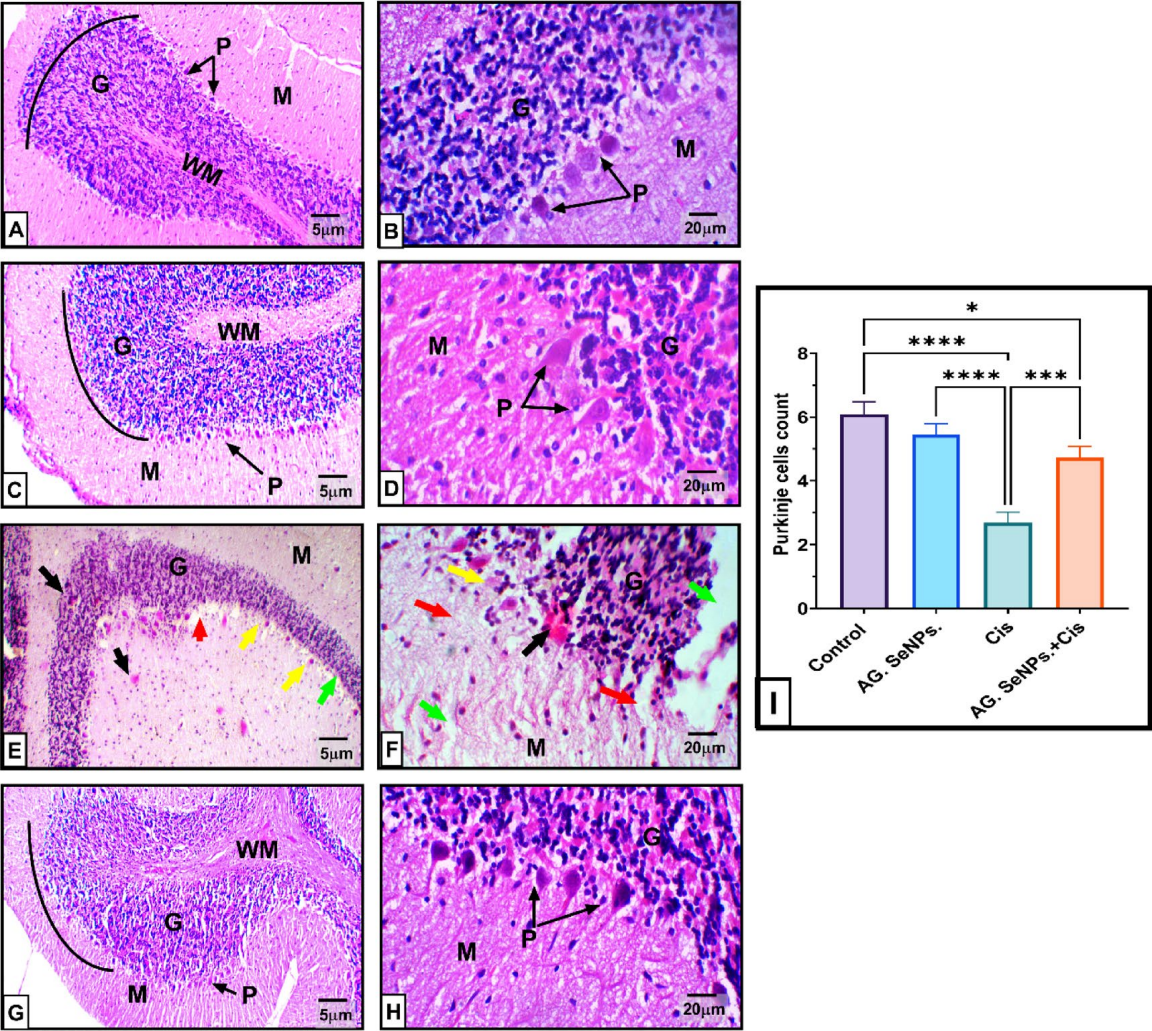
Photomicrographs of H&E-stained cerebellar tissue across experimental groups. (**A**, **B**) Control and (**C**, **D**) AG-SeNPs groups: These sections demonstrate the typical three-layered architecture of the adult cerebellar cortex, consisting of the superficial molecular layer (M), the monolayer of Purkinje cells (P), and the deep, highly cellular granular layer (**G**). The morphology appears organized and intact. (E&F) Cisplatin (Cis) group: These sections reveal obvious neurotoxic damage. Notable features include nuclear pyknosis (yellow arrow), vacuolized neuroplasm (green arrow), and areas of significant necrosis with hypereosinophilia (black arrow). There is also a localized loss of Purkinje cells, characteristically leaving behind “empty space” (red arrow). (G&H) Cis + AG-SeNPs group: These sections show substantial structural restoration. While a few degenerated cells with pyknotic nuclei (black arrow) are still visible within the Purkinje cell layer (P), the overall organization of the granular (**G**) and molecular (M) layers remains relatively normal, indicating a protective effect of the nanoparticles. (**I**): Quantitative representation showing the mean Purkinje cell count per linear millimeter across the experimental groups; Data are expressed as mean ± S.E. (*n* = 6). *, **Significant change at *P* < 0.05 and *P* < 0.01 respectively.‎‎ Magnification: Left panels are shown at X100; right panels are shown at X400.

Control and AG-SeNPs Groups histopathological examination of the cerebellar tissue revealed a well-preserved trilaminar cortical architecture. The superficial molecular layer appeared compact and lightly pigmented, containing nerve fibers and dispersed stellate and basket cells. Beneath this, the Purkinje cell layer exhibited a characteristic monolayer of large, pyriform (pear-shaped) cell bodies with distinct nuclei. The innermost granular layer, adjacent to the cerebellar white matter, was composed of densely clustered granule cells characterized by small, heterochromatic, and darkly stained nuclei. Cisplatin Group, in contrast, showed significant neurotoxic alterations. There was a noticeable depletion of the Purkinje cell layer, with remaining cells displaying acidophilic cytoplasm and karyolysed nuclei. The presence of activated, giant, irregular glial cells (gliosis) further indicated significant tissue damage resulting from platinum-induced neurotoxicity. Co-treatment with AG-SeNPs and Cisplatin markedly preserved the structural integrity of the cerebellar folia. While much of the architecture was restored, some granule cells still exhibited pyknotic, basophilic characteristics. However, several Purkinje cells maintained normal morphology, indicating that the ‎combination of Arabic gum-coated selenium nanoparticles with Cisplatin significantly attenuated the neurotoxic impact compared to the Cisplatin-only group. These changes are clearly illustrated in Fig. 7.

**Table 2:** Semi-quantitative histopathological scores of cerebellar damage.

Group	Edema	Vacuolation	Pyknosis
Control	0.84±0.14	0.88±0.11	0.96±0.15
AG. SeNPs	0.88±0.15	0.96±0.14	1.04±0.15
Cis	3.44±0.13 a****	3.280±0.14 a****	3.52±0.1 a****
AG. SeNPs.+Cis	1.9±0.25a*, b**	1.583±0.15b****	2.17±0.22 a**,b**

Data are expressed as mean ± S.E. (*n* = 6). *, **, ****Significant change at *P* < 0.05 and *P* < 0.01, *P* < 0.0001 respectively. a indicating Control; b indicating Cis group).

### Immunohistochemical investigations

#### GFAP protein expression

**Fig. 8:**
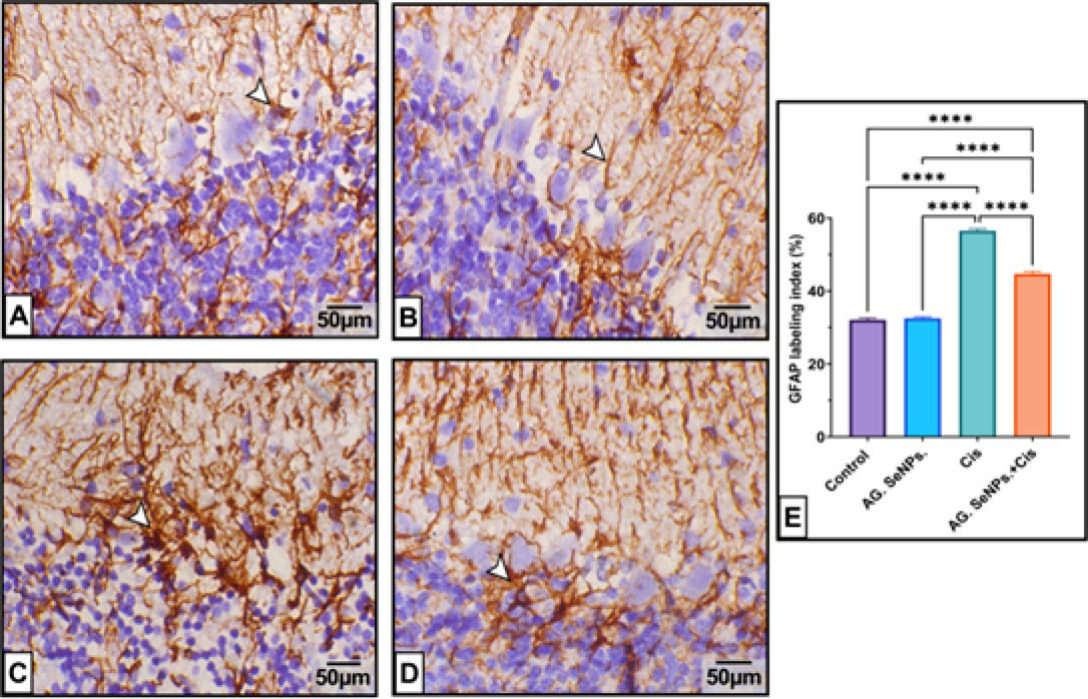
Effect of Cis and/or AG. SeNPs. on the immuno-expressional level of GFAP protein in rat cerebellar tissue of different groups. (**A**) Control and (**B**) AG. SeNPs. groups showed neural filaments and small astrocytes with short processes and a mild brown stain in the granular and molecular layers. (**C**): Cis group showed significant GFAP upregulation in different cerebellar layers. (**D**) AG. SeNPs. + Cis cotreatment significantly reduces GFAP expression (arrowheads). (**E**) GFAP labeling index; Data are expressed as mean ± S.E. (*n* = 6). ****Significant change at *P* < 0.0001. Scale bar: 50 μm.

Immunohistochemical analysis revealed mild GFAP expression in cerebellar tissues from both control and AG. SeNps treated animals. Meanwhile, Cisplatin administration significantly upregulated GFAP protein expression compared to the control group (*P* < 0.001). However, cotreatment with AG. SeNPs. markedly downregulated GFAP protein expression (42.515%, *P* < 0.05) compared to the cisplatin-treated group (Fig. 8).

#### NFL protein immunoexpression

**Fig. 9:**
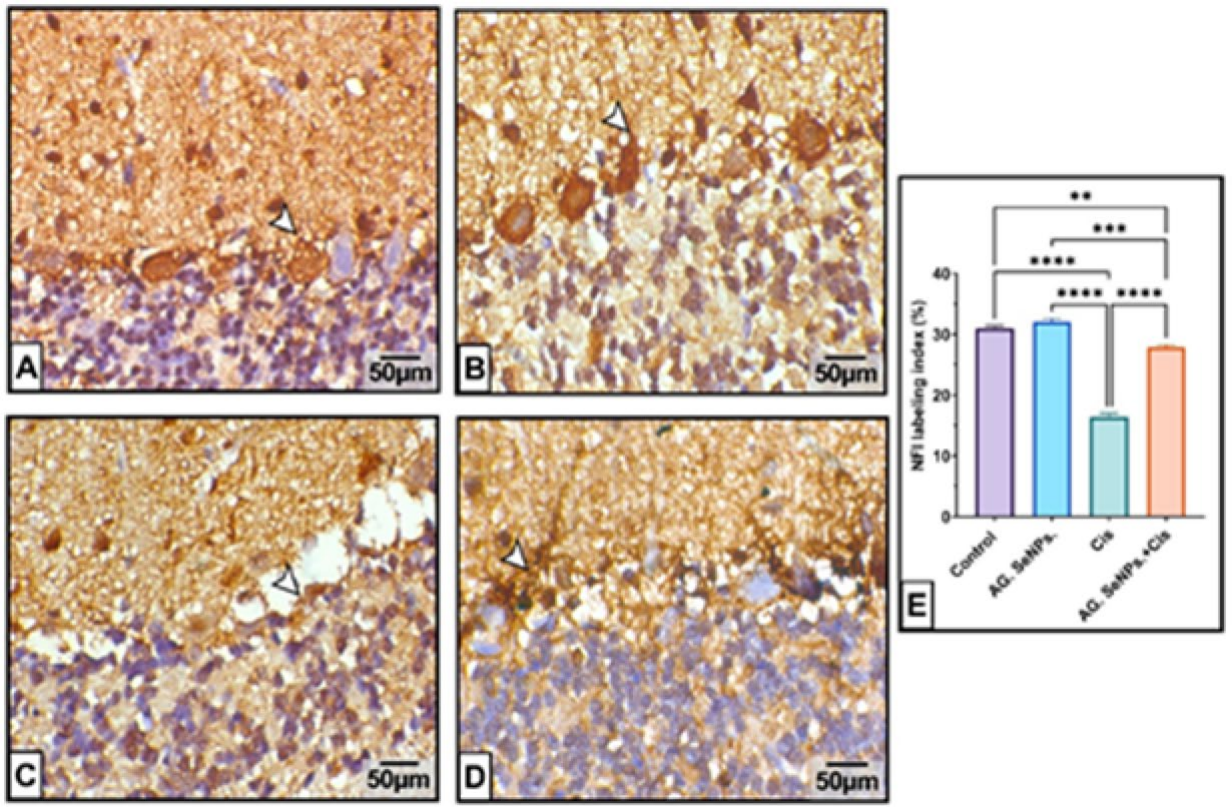
Effect of Cis and/or AG. SeNPs. on the immuno-expressional level of NFL protein in male rat cerebellar tissue of different groups. (**A**): The Control and (**B**) AG. SeNPs. treated animals exhibit normal NFL expressions in Purkinje cells and molecular layers. (**C**) cisplatin administered animals show marked NFL intensity downregulation in the Purkinje cell layer. (**D**) AG. SeNPs. treatment further enhances NFL expression in combining group. (**E**) NFL labeling index; Data are expressed as mean ± S.E. (n=6). **, ***, **** Significant change at P < 0.01, P < 0.001, P < 0.0001 respectively. (arrowheads). Scale bar: 50 µm.

Immunohistochemical analysis revealed mild NFL intensity expression in cerebellar tissues from control and AG. SeNps. treated animals. Cisplatin administration remarkably decreased NFL expression compared to the control group (*P* < 0.001). However, treatment with both AG. SeNPs. and cisplatin significantly enhanced NFL intensity (96.264%, *P* < 0.05) as represented in Fig. 9.

### Ultra structure examination

**Fig. 10:**
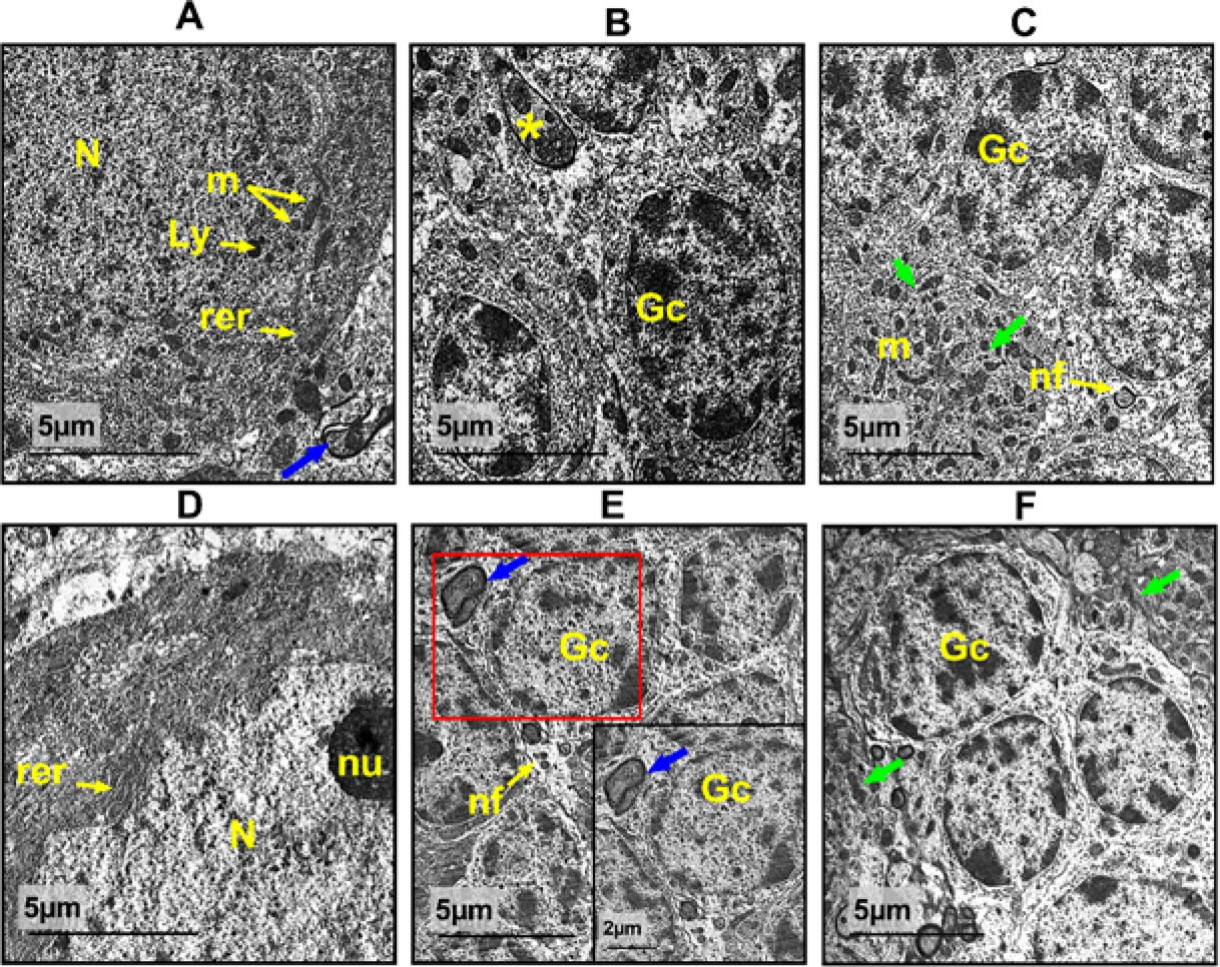
Electron micrographs of cerebellar cortex sections from control group (**A–C**) and AG. SeNps., (**D**–**F**) group. Exhibit typical Purkinje cells containing a standard eukaryotic nucleus (N). The cytoplasm contains numerous rough endoplasmic reticulum (rer), few lysosomes (Ly) and granulocytes (Gc), is abundant in mitochondria (m), and the granular layer exhibits a multitude of myelinated nerve fibers (nf), neural axons (*) and pre- and post-synapses (blue arrow) and intact neuropil (green arrow). Scale bar 5 μm, insets 2 µm.

Figure 10A–C, showed the ultrastructural analysis of the control group’s cerebellar cortex with an intact pyriform cell with a distinct eukaryotic nucleus and a sizable electron-dense nucleolus. It was revealed that the cytoplasm of this cell was abundant in RER, secretory granules, and both elongated and spherical mitochondria. Normal granulocytes with primarily peripheral central euchromatin were visible in the granular layer. Following the administration of SeNps coated with Arabic gum, Purkinje cells with a euchromatic nucleus, abundant RER, Golgi apparatus, and mitochondria of various sizes were shown to be quite typical. The granular layer exhibited normal organization as that of the control as shown in Fig. 10D–F.

**Fig. 11:**
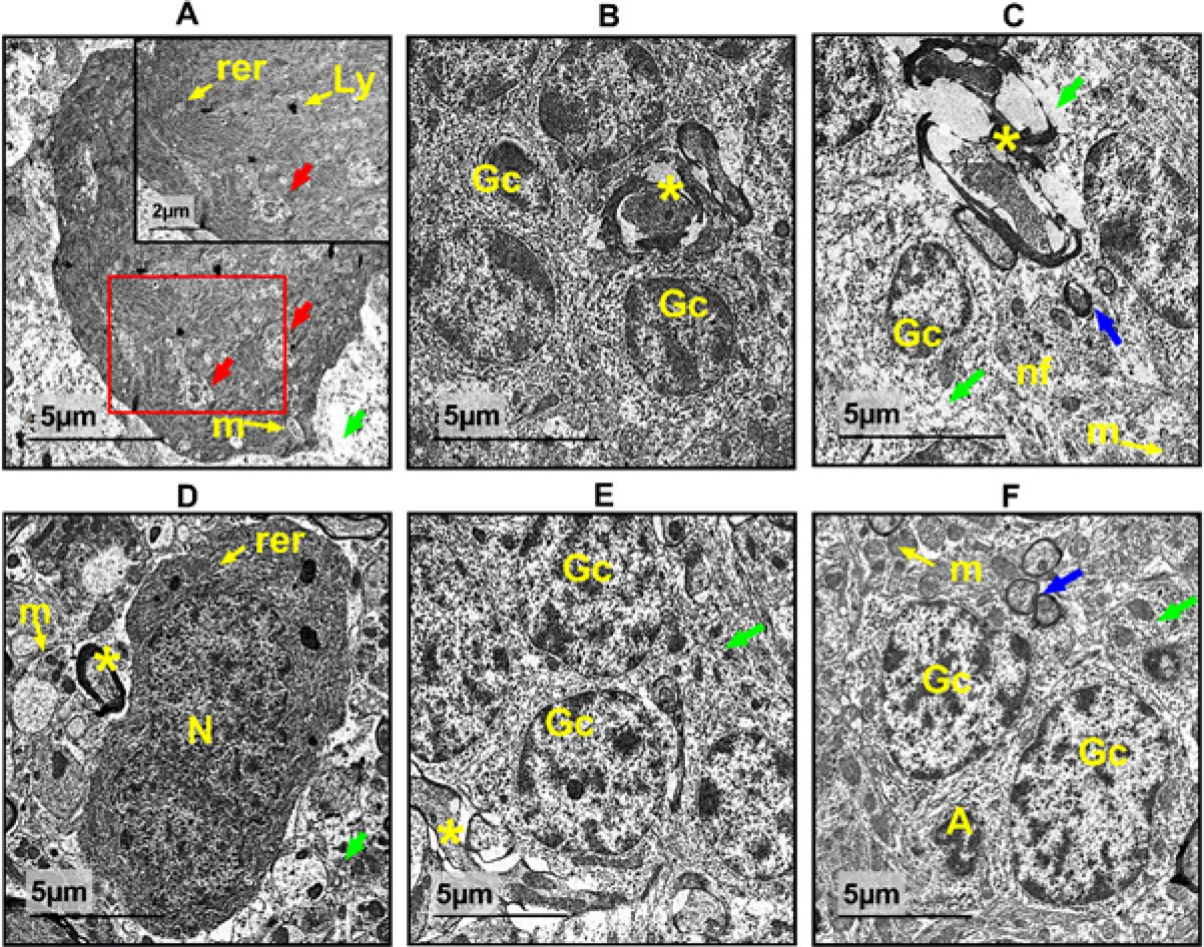
Electron micrographs of cerebellar cortex sections from cisplatin treated groups (**A**–**C**) and Cis + AG. SeNps. Group (**D**–**F**). The cisplatin sections exhibit Purkinje cells with pyknotic nuclei (N), dilatation of the rough endoplasmic reticulum (rer), vacuolated nucleoplasm (red arrow), swollen mitochondria (m), degenerated neuropil (green arrow). Splitting and break down of the myelin sheath result in the appearance of myelinated nerve axons (*) and neural filaments (nf), and the granular layer shows destroyed granulocytes with pyknotic nuclei and an invaginated nuclear envelope (Gc). (Cis + AG. SeNps.) Combined group showed quite intact Purkinje cells with normal nucleus (N), dilated rough endoplasmic reticulum (rer), granulocytes (Gc), normal axons (*). Pre-normal mitochondria (m), neuropil (green arrow) and pre- and post-synapses (blue arrow) while still suffering from apoptosis (**A**). Scale bar 5 μm, insets 2 µm.

Figure 11A–C, showed that Purkinje cells displayed apoptotic changes, such as dark atrophied pyknotic nuclei and dimpled envelopes, indicating the cisplatincerebellar damage. Its cytoplasm showed signs of vacuolation, RER dilatation, and injured nerve fibres with broken myelin sheaths in the granular layer. Additionally, as illustrated in Fig. 11D–F, certain brain granulocytes were demonstrated to be degenerated with pyknotic nuclei and invaginated nuclear envelopes in a different focus. On the other hand, combined treatment with both cisplatin and AG. SeNPs. revealed a marked recovery in all cerebellar cortex with quite intact Purkinje cells with a well-defined cytosol, pre-normal neuropil with active granulocytes and myelinated nerve fibers.

### Molecular docking of Kaempferol with the recognition site of TNF-α, Caspase-3 and IL-6

Docking of Kaempferol with the recognition site of TNF-α (tumor necrosis factor alpha) (PDB ID: 2AZ5) showed conventional hydrogen bond interaction with TYR A:151, GLY-121 and TYR B:151,moreover there are van der Waals force with TYR-59, GLN B :61 and GLN A :61. Kaempferol’s combined affinity for TNF-α was − 7.4 kcal/mol53. The Gly, Ser-120, Phe, and Trp residues in the contacts with kaempferol provided van der Waals interactions when it docked with the Caspase-3 recognition site (PDB ID: 1NMS). Furthermore, kaempferol formed three hydrogen bonds with the residues Arg-207, His-121, and Tyr-204 in order to connect with Caspase-3.and a negative donor-donor relationship with Gln-161.When kaempferol was exposed to Arg-207 and Tyr-204, pi-cation and pi-pi T-shaped interactions occurred^53^. Moreover, the combination affinity of Kaempferol on Caspase-3 was − 8 kcal/mol. Docking of Kaempferol with the recognition site of IL-6 (PDB ID: 1ALU) showed conventional hydrogen bond interaction with ARG A: 30, moreover there are van der Waals force with LEU A:33, GLN A: 175 and ARG A: 182^54,55^. The combination affinity of Kaempferol on IL-6 was − 6.5 kcal/mol as shown in Table 3.

**Table 3:** Docking of Kaempferol with the recognition site of TNF-α, Caspase-3 and IL-6.

Compound	Target protein (ligand)	PDP ID	Binding affinity (kcal/mol)	Visualization
Kaempferol	TNF-α	2AZ5	-7.4	
	Caspase-3	1NMS	-8	
	IL-6	1ALU	-6.5	

### Molecular docking of Luteolin with the recognition site of TNF-α, Caspase-3 and IL-6

Docking of Luteolin with the recognition site of IL-6 (PDB ID: 1ALU). The combination affinity of Luteolin on IL-6 was − 7 kcal/mol. The hydroxyl group of the ligand establishes hydrogen bonds with Arg179, Gln175, and Asp34. Hydrophobic residues Leu178, Arg30, and Leu30 create a robust stacking interaction with the hydrophobic ligand ring to enhance binding stability^56^. Docking of Luteolin with the recognition site of TNF-α (PDB ID: 2AZ5) showed van der Waals force with TYR C: 151 and GLN C: 61. The combination affinity of Luteolin on TNF-α was − 7.8 kcal/mol^57^. Binding mode analysis of luteolin docking with Caspase-3 (PDB ID :1NMS).yielded a good binding free energy () of −6.6 kcal/mol, predicting a good inhibitory potential. The structural stability of the binding pose was confirmed by good RMSD value of 1.6ÅThe 2D diagram refer to pi-pi t shaped interaction with TRP A :206 and van der Waals force with PHE A :256, ARG A:207 ASN A:208 TRP A :214 PHE A:250 and SER A:2511NMS This specific PDB structure represents human Caspase-3. Successful docking suggests Luteolin can physically fit into the enzyme’s regulatory or active sites to modulate its function as presented in Table 4.

**Table 4:** Docking of Luteolin with the recognition site of TNF-α, Caspase-3 and IL-6

Compound	Target protein (ligand)	PDB ID	Binding affinity (kcal/mol)	Visualization
Luteolin	IL-6	1ALU	-7	
	TNF-α	2AZ5	-7.8	
	Caspase-3	1NMS	-6.6	

### Molecular docking of quercetin with the recognition site of IL-6, Caspase-3 and TNF-α

The current study examined the interactions between quercetin and caspase-3 (PDB ID:1NMS), uncovering a variety of notable binding interactions. The quercetin molecule demonstrates many interactions with critical amino acid residues, including van der Waals forces with tyrosine TYR: 197 and ARG: 164. Furthermore, pi-anion interactions were detected with glutamic acid GLU: 124 and proline PRO: 201, while pi-alkyl interactions were identified with VAL: 266 and VAL: 826^58^. These findings emphasize the intricate characteristics of the binding site and show the potential of quercetin as a regulator of caspase-3 function. Quercetin’s combined affinity for caspase-3 was − 8.2 kcal/mol. Quercetin docking with the IL-6 recognition site (PDB ID: 1ALU). Quercetin’s combined affinity for IL-6 was − 6.8 kcal/mol^55^. Through IL6 target proteins ARG-30, LEU-33, GLN-175, LEU-178, and ARG-179, quercetin stably binds to the IL6 active site^59^. Docking of Quercetin with the recognition site of TNF-α (PDB ID: 2AZ5) showed Combining with the amino acid residues TYR: 119, LEU: 120, TYR:59 and SER: 60^60^. The combination affinity of Quercetin on TNF-α was − 8.8 kcal/moL. The molecular docking investigation of the query ligand with Tumor Necrosis Factor (TNF) identified critical interactions that may enhance its binding affinity and possible inhibitory efficacy. The ligand demonstrated van der Waals interactions with residues TYR B:59, GLY B:121, GLY B:122, VAL B:123, and VAL D:123, signifying weak yet stabilizing forces inside the binding pocket. Furthermore, Pi-Sigma interactions were noted, indicating potential electronic contributions to ligand stability. Significantly, Pi-Alkyl interactions with LEU B:57 underscore the existence of hydrophobic contacts that might enhance ligand anchoring. These interactions indicate that the ligand may proficiently attach to TNF, possibly disrupting its activation, as shown in Table 5‎.

**Table 5:** Docking of Quercetin with the recognition site of IL-6, Caspase-3 and TNF-α

Compound	Target protein (ligand)	PDB ID	Binding affinity (kcal/mol)	Visualization
Quercetin	Caspase-3	1NMS	-8.2	
	IL-6	1ALU	-6.8	
	TNF-α	2AZ5	-8.8	

### Molecular docking of ferulic acid with the recognition site of IL-6, Caspase-3 and TNF-α

Binding mode analysis of ferulic acid with Caspase-3 (PDB ID: 1NMS). yielded a favorable binding free energy $\:\left(\varDelta\:G\:\right)$ of −5.4 kcal/mol, predicting a good inhibitory potential. The structureal stability of the binding pose was confirmed by RMSD value of 1.5 Å. There are a lot of interactions shown in pi-pi between the aromatic ring of ferulic acid and the residue TRPA :206. Moreover, the ligand is stabilized by a van der Waals forces including ASN A:208, ARG A : 207, PHE A : 256, SER A:251, PHE A :250 and SER A :249.the presence of these multiple non covalent interactions suggests a high degree of complementary between the ligand and the enzymes binding pocket, supporting the potential of ferulic acid to modulate caspase-3 activity. Binding mode analysis of ferulic acid with IL-6 (PDB ID: 1ALU). yielded a good binding free energy $\:\left(\varDelta\:G\:\right)$ of −5 kcal/mol, predicting a good inhibitory potential. The structural stability of the binding pose was confirmed by RMSD value of 1.8 Å. The pi-anion interaction, a electrostatic interaction, is formed between the aromatic ring of ligand and the carboxylate group of GLU A: 93. This interaction is crucial for anchoring the molecule within pocket. van der Waals forces with residues ASN A :63, PRO A :65, VAL A:96 and PRO A: 139. The presence pf TYR A:97 suggests additional hydrophobic stabilization. Binding mode analysis of ferulic acid with IL-6 (PDB ID: 1ALU). yielded a good binding free energy $\:\left(\varDelta\:G\:\right)$ of −7.2 kcal/mol, predicting a good inhibitory potential. The structural stability of the binding pose was confirmed by good RMSD value of 1.3 Å. This complex is the formation of conventional hydrogen bond between the hydroxyl group of ferulic acid and the amino acid residue GLY B: 122. This specific interaction is critical for the directional orientation and anchoring of the ligand. Van der Waals interactions with several surrounding residues across both chains of the protein, including TYR B :119, TYR B :59, ILE Bl58, LEU B : 57, GLY B:121, ILE A : 155 and LEU A : 57. This interactions both chain A and chain B suggests that ferulic acid may potentially contributing to the anti-inflammatory activity observed in the study (Table 6).

**Table 6:** Docking of ferulic acid with the recognition site of IL-6, Caspase-3 and TNF-α.

Compound	Target protein (ligand)	PDB ID	Binding affinity (kcal/mol)	Visualization
Ferulic acid	Caspase-3	1NMS	-5.4	
	IL-6	1ALU	-5	
	TNF-α	2AZ5	-7.2	

## Discussion

Cisplatin-induced central neurotoxicity is a significant concern. Cisplatin, a widely used chemotherapeutic agent, cannot effectively reach the brain due to the blood-brain barrier^61^. Since the barrier’s morpho-functional maturation takes place during the 21 st day of the rat’s postnatal life, this is especially problematic during brain development when it is not fully developed^62^. Furthermore, cisplatin targets specific neurons, impacting potassium channel function and increasing their excitability. Animal studies have confirmed that cisplatin exposure can lead to brain damage. Potential therapeutic chemicals can be found in considerable quantities in medicinal plants. Their varied pharmacological qualities, including as antibacterial, antioxidant, anti-inflammatory, and anticancer activities, have been the subject of extensive research. Furthermore, continuing research on plant phytochemicals, including terpenes, alkaloids, flavonoids, and phenolic compounds, is opening up exciting new possibilities for improvements in environmental sustainability, nutrition, and medicine^63^.

Specific plants of the Acacia genus are the source of AG^64^.The transport of pharmaceuticals across the blood-brain barrier presents a significant challenge. However, selenium nanoparticles (SeNPs) have emerged as promising candidates for drug delivery, particularly in the context of neurodegenerative disorders^65^. Numerous studies have demonstrated the superior bioavailability, enhanced antioxidant activities, and reduced toxicity of selenium nanoparticles compared to other selenium-containing compounds. Moreover, selenium nanoparticles possess free radical scavenging capabilities and can ameliorate behavioral aberrations and neurochemical changes. In addition, they have the potential to improve memory impairment, making them a promising therapeutic option for neurodegenerative diseases^66^. The aim of this study was to evaluate how these nanoparticles coated with Arabic gum can modulate and mitigate cis platin induced cerebellar damage in adult male albino rat.

According to the current study results, the phytochemicals are extracted as flavonoids compounds, Naringin Quercetin, Kaempferol, Luteolin and Catechin and phenolic Acid compounds, Syringenic, P-Coumaric, Cinnamic, Caffeic, Gallic and Ferulic on Arabic gum. These results align with^67^who reported the same phenolic and flavonoid compounds. Flavonoids and phenolic acids function as antioxidants by scavenging free radicals and mitigating free oxidative stress. This safeguards cells and tissues from harm. These compounds work by donating electron for free radicals stabilizing them and preventing them from cellular damage^68^.

The findings of the present study indicate that cisplatin toxicity on antioxidant enzyme activity and free radicals’ production by significantly decreasing levels of GSH, CAT, and SOD, while increasing levels of 8-OHdG, MDA and H_2_O_2_. This imbalance may result in considerable harm to cellular constituents, encompassing lipids, proteins, and DNA^69^. Over 100 alterations in DNA caused by Reactive oxygen species have been documented. The activities of superoxide dismutase (SOD), catalase (CAT), glutathione (GSH), malondialdehyde (MDA), hydrogen peroxide (H₂O₂), and 8-hydroxydeoxyguanosine (8-OHdG) function as markers of the oxidative status in both tissue and blood. Furthermore, the increased levels of MDA, H₂O₂, and 8-OHdG indicate a substantial generation of free radicals. These markers are essential for comprehending the mechanisms of oxidative stress and its effects on cellular health. Moreover, these markers indicate that elevated oxidative stress levels can activate the apoptotic cell death pathway, resulting in neurodegeneration^70,71^.

After administrating of selenium nanoparticles coated with Arabic gum to rats there was a significantly increased in the GSH, CAT, and SOD levels, while decreased in the 8-OHdG, MDA and H_2_O_2_ levels compared to cisplatin animals. These results were in parallel with previous studies, which explained that selenium nanoparticles and Arabic gum caused regression in production of free radicals and increased in production of antioxidant activity such as SOD, CAT and GSH^72^. Further study revealed selenium nanoparticle’s protective effect on improvement neurological disorders as anti-inflammatory and antioxidant^73^ and improvement cerebellum injuries^73^.

In the present study cisplatin group showed increasing in the caspase 3 expression which align with the known mechanism of cisplatin induced apoptosis^74^. Our findings demonstrate that selenium nanoparticles coated with Arabic gum significantly reduced the expression of Caspas-3 in neuronal cells exposed to cisplatin. Cisplatin treatment led to a marked increase in Caspas-3 expression, indicative of DNA damage and activation of the apoptotic pathway. This aligns with previous research demonstrated that caspases especially caspase-3, are activated during apoptosis and are essential for the execution of cell death. The down regulation of Caspase-3 by the selenium nanoparticles coated with Arabic gum suggests a potential mechanism for their neuroprotective effects. This is aligned with previous study documented that therapeutic potential effect of selenium nanoparticles in central nervous system^75^. Caspase 3 is integral to the apoptosis triggered by cisplatin, chemotherapeutic agent frequently employed in the treatment to diverse malignancies^76^.

Cisplatin in the current results recognized significant increase in p53 expression because of its DNA-damaging effects as reported by^77^. Cisplatin treatment led to a marked increase in P53 expression, indicative of DNA damage and activation of the apoptotic pathway. This aligns with previous research demonstrating that chemotherapy can induce P53 mutations^78^. Our findings demonstrate that selenium nanoparticles coated with Arabic gum significantly reduced the expression of P53 in neuronal cells exposed to cisplatin. The regulation of p53 by selenium nanoparticles suggests that chemotherapy can induce p53 mutations, leading to alterations in the apoptotic pathway and potentially affecting the overall cellular response to treatment. This modulation indicates a promising protective effect of selenium nanoparticles coated with Arabic gum against the DNA damage and apoptosis typically induced by cisplatin. The downregulation of P53 by the selenium nanoparticles coated with Arabic gum suggests a potential mechanism for their neuroprotective effects. Since P53-dependent apoptosis is a common pathway in neuronal cell death, inhibiting its activation could contribute to the observed reduction in neuronal cell death^79^.

The present investigation showed that rats administered cisplatin show a marked decrease in serum neurotransmitter serotonin, and dopamine levels. The findings closely resembled the preliminary results indicating cisplatin-induced neurotoxicity, with male rats exhibiting greater weight loss, extended heat latency, and diminished motor nerve conduction velocity (MNCV)^79^. Cisplatin induces oxidative stress, which can damage neurons and affect the synthesis and release of neurotransmitters like serotonin and dopamine^80^.

Our findings demonstrated that SeNPs significantly elevated the serotonin levels in prefrontal cortex tissue compared to control group. This result is consistent with another study that demonstrated the antioxidative qualities of the natural components (phenolic acid and flavonoids) present in Arabic gum. Disorders of the central nervous system, such as Parkinson’s, Alzheimer’s, and traumatic brain traumas, may be treated using SeNPs. They have excellent anti-inflammatory and antioxidant capabilities, as well as specific drug delivery systems in the brain. By lowering neuroinflammation and possibly oxidative stress^75^. Furthermore, when compared to those treated with cisplatin alone, the administration of selenium nanoparticles coated with Arabic gum raises serum levels of neurotransmitters (dopamine and serotonin). Our results were consistent with recent research showing that giving adult male albino rats Arabic gum and selenium nanoparticles increased their neurotransmitters^81,82^.

The inflammatory markers TNF-α, IL-10, and IL-6 were significantly higher in the cis animal group than in the control group, according to the current study. Preclinical studies have shown that cisplatin intoxication causes an inflammatory response in male mice’s cerebellar cortex^83^. Furthermore, the expression of pro-inflammatory and inflammatory markers was significantly elevated in the cisplatin-treated cohort. The outcomes were consistent with previous research, showing that cisplatin can cause the production of the cytokines TNF-α, IL-6, and IL-10 in mouse brains^84^. Pro-inflammatory cytokines are critical inflammatory mediators that significantly contribute to neuroinflammation^85^. In a cerebellar culture model of neuroinflammation, proinflammatory cytokines contribute to demyelination and axonal damage. Furthermore, preclinical studies have linked neuroinflammation and the release of pro-inflammatory cytokines to cognitive impairment, including that observed in Alzheimer’s disease. Considered a natural substance, Arabic gum contains numerous antioxidants that have been successful in reducing the inflammatory effect and causing the cytokines IL-6, IL-10, and TNF-α to regress^86^. Selenium nanoparticles have an anti-inflammatory impact, coating them with Arabic gum is a simple procedure that improves their stability and biocompatibility. These results were consistent with ours, which showed that groups treated with selenium nanoparticles coated with Arabic gum experienced a considerable decline in cytokines.

In this study, the interaction between flavonoids (kaempferol, luteolin, quercetin and Feurlic acid) and their target protein as TNF- α, IL-6 and Caspase-3 was further reflected by the molecular docking which provided some information about their possible anti-inflammatory and pro-apoptotic effects. Kaempferol showed significant binding affinity with targets protein (TNF- α = −7.4 Kcal/mol, IL-6 = −6.5 Kcal/mol and Caspase-3 = −7.4 Kcal/mol), Luteolin docked with (TNF- α = −7.8 Kcal/mol, IL-6 = −7 Kcal/mol and Caspase-3 =−6.6 Kcal/mol), Quercetin docked with same target protein (TNF- α = −8.8 Kcal/mol, IL-6 = −6.8 Kcal/mol and Caspase-3 = −8.2 Kcal/mol) and Feurlic acid docked with (TNF- α =−7.2 kcal/mol, Il-6 = −5 kcal/mol and Caspase-3= −5.4 kcal/mol). These docking results complement with the biochemical analysis, where kaempferol, quercetin, luteolin and Feurlic acid revealed significant inhibition of inflammatory markers, align with silico predications.

In the present study, damage of cerebellum was found in adult male albino rats intoxicated with cisplatin. Exposure of cerebellum to cisplatin causes degeneration in cerebellum layers (Molecular, Purkinje, and granular layer), decreasing in number of Purkinje cell, necrotic and pyknotic granular cells. This finding aligned with prior studies on cisplatin’s effects on the cerebellum cortex structure and locomotor activity in rats, emphasizing oxidative stress-related modifications and histological changes, including vacuolations, reduced thickness, and bleeding^87^. Furthermore, it revealed gliosis, Purkinje cell degeneration, multifocal shrinkage, and neuronal damage combined with Purkinje layer neuronal degeneration. Cisplatin causes oxidative stress and histological changes include granular cell disintegration, disorganisation, and degeneration, according to preclinical research. Purkinje cells demonstrated irregular pyknotic nuclei, cytoplasmic vacuolation, and mitochondrial damage^88^. These findings indicated that selenium nanoparticles coated with Arabic gum can mitigate the histopathological alterations caused by cisplatin and enhance cerebellar bioactivities when administered concurrently with cisplatin, demonstrating the synergistic effect of selenium nanoparticles coated with Arabic gum against cisplatin toxicity.

In this study, immunohistochemical analysis revealed a marked increase in glial fibrillary acidic protein (GFAP) expression in the cisplatin-treated groups, indicating astrocyte activation. In contrast, treatment with selenium nanoparticles coated with Arabic gum significantly reduced GFAP expression compared to the cisplatin group. GFAP is a type III intermediate filament protein encoded by the GFAP gene and is predominantly expressed by astrocytes in the central nervous system (CNS). It plays a vital role in maintaining the structural integrity and functional stability of astrocytes, which are essential for neuronal support and homeostasis^89,90^. Moreover, GFAP is extensively utilized as a biomarker in neuroscience research and clinical diagnostics. Increased concentration of GFAP in CSF fluid or blood signify astrocyte activation or damage and are utilized to diagnose or monitor aliments such as traumatic brain injury, multiple sclerosis, and specific neurodegenerative disorders. This result was parallel with previous studies which demonstrated the rise of GFAP positive cells caused by cisplatin that is frequently employed, has the potential to cause chemotherapy-induced peripheral neuropathy (CIPN), a condition that entails neuroinflammation and neuronal injury. Additionally, GFAP expression significantly decreases after selenium nanoparticles coated with Arabic gum are administered, demonstrating the antioxidant potential of these particles against cisplatin neurotoxicity. This result was in line with recent research that demonstrated that by lowering oxidative stress and safeguarding brain cells, selenium nanoparticles coated with Arabic gum can decrease this expression. Furthermore, these results showed that the antioxidant properties of Arabic gum may be used to inhibit the production of GFAP by coating selenium nanoparticles with it. This conclusion was consistent with the previous finding that the anti-inflammatory, anti-oxidative, and neuroprotective qualities of Arabic gum and selenium nanoparticles might be attributed to them^91^. Thus, immunohistochemical labelling of the rat cerebellar cortex reflects the antioxidant properties of Arabic gum and selenium nanoparticles, potentially averting the negative effects of cisplatin toxicity^92^.

In the current results, immunohistochemical staining of Neurofilament light (NFL) intensity showed significant reducing in Neurofilaments in cisplatin treated groups. On the other hand, selenium nanoparticles coated with Arabic gum treated group showed elevation in NFL compared to cisplatin treated group. This finding aligned with prior studies on cisplatin’s effects that can cause a reduction in neurofilament light (NFL) is refer to degenerative neurons^93^. Neurofilaments are Neuroskeleton found mostly in axons. Axon size, caliber and shape are all regulated by these structures and they also serve as structural supports^94^.it is a member of family of intermediate filaments, neurofilaments consist of a triplet of three subunits : the light chain (NF-L), the medium chain (NF-M) and the heavy chain (NF-H). Axonal damage and neuronal death are indicated by the release of neurofilament proteins into cerebrospinal fluid (CSF). Following axonal damage in the central nervous system (CNS). Among these subtypes, NF-L has received the greatest amount of research attention^94^. Other studies showed that Neurofilaments are in the cytoplasm of neurons all conditions resulting in neuronal and axonal injury can elevate the amount of these proteins in cerebrospinal fluid (CSF). NF-l levels have served as a biomarker for axonal injury in animal studies for decades. Our results, in line with studies, demonstrated that antioxidants can influence NF-L. It has a beneficial effect on neurofilament light (NFL) levels, which are frequently linked to neuronal injury and neurodegenerative disorders^95^ We found improvements in selenium nanoparticles coated with Arabic gum treated group compared to cisplatin treated group. Consequently, the immunohistochemical staining of the rat cerebellar cortex demonstrates the antioxidant qualities of selenium nanoparticles coated with Arabic gum, which may avert the negative consequences brought on by cisplatin toxicity.

In the current work, a transmission electron microscope (TEM) was used to evaluate structural alterations in the cerebellum of rats given cisplatin. Because there were less Purkinje cells in the Purkinje layer of the cisplatin-treated group, the cerebellar cortex of the group treated with selenium nanoparticles coated with Arabic gum had neurodegenerative traits. histopathological changes in the cerebellar cortex as well. This damage includes cellular necrosis in the molecular layer, aberrant pyknotic nuclei in Purkinje cells, and granular cell disintegration and degeneration^96^. In this study, the group that received treatment with selenium nanoparticles coated with Arabic gum lessened the degree of cerebellar changes observed in the group that received cisplatin. It’s interesting to note that the combination of cisplatin and selenium nanoparticles coated with Arabic gum significantly improved the cerebellum’s ultrastructure, which had been destroyed by cisplatin treatment alone^97^. A notable alteration in neurodegeneration was observed at the ultrastructure level in the cisplatin group, indicating that neuronal subcellular death could contribute to the development of the histopathological alterations documented here.in addition, the current results suggest that DNA damage in neurons after cisplatin exposure and cell arrest may be the underlying cause of the apparent cell death and necrosis^98^. Neuropathy may develop because of DNA damage and Schwann cell corruption brought on by cytotoxic cisplatin’s association with cell death, mitochondrial cristae, and myelinated axon loss. The chemotherapeutic dosage of cisplatin is bio transformed into active forms, specifically harmful metabolites, through hydrolysis, resulting in mitochondrial damage^83^.

## Conclusion

The study concluded that selenium nanoparticles coated with Arabic gum demonstrated significant protective and therapeutic effects against cisplatin- induced cerebellar damage in adult male albino rats. The administration of these nanoparticles modulated and reduced the neurotoxic effects of cisplatin, indicating their potential as a beneficial intervention. The findings suggest that selenium nanoparticles coated with AG could serve as a promising approach to mitigate the adverse impacts of cisplatin on the cerebellum, improving the overall neuroprotection during chemotherapy treatments. The present study is subject to several limitations that should be acknowledged. Comprehensive evaluations of stability and shelf life under physiological conditions were not performed, and the absence of additional control groups restricted thorough characterization of the formulation. Moreover, the use of a single acute cisplatin dose, together with the indirect evaluation of blood–brain barrier penetration, limits the extent to which the findings can be extrapolated to clinical settings. The lack of behavioral assessments and in vitro validation also constrained the interpretation of functional outcomes and underlying mechanisms. Additionally, the relatively high polydispersity index of the AG-SeNPs reflects particle size heterogeneity, likely attributable to the green synthesis approach. Although the nanoparticles demonstrated acceptable stability and consistent biological effects, further optimization of the synthesis process to achieve greater size uniformity would enhance the robustness and translational potential of the formulation.

## Data Availability

Data will be available when requested from the corresponding author.

## References

[1] Sokolov, A. A., Miall, R. C. & Ivry, R. B. The cerebellum: adaptive prediction for movement and cognition. *Trends Cogn. Sci.* **21**, 313–332 (2017). 28385461 10.1016/j.tics.2017.02.005 PMC5477675

[2] Hirano, T. Motor control mechanism by the cerebellum. *Cerebellum* **5**, 296 (2006). 17134993 10.1080/14734220600776387

[3] Florea, A. M. & Büsselberg, D. Cisplatin as an anti-tumor drug: cellular mechanisms of activity, drug resistance and induced side effects. *Cancers (Basel)*. **3**, 1351–1371 (2011). 24212665 10.3390/cancers3011351 PMC3756417

[4] Wick, A. et al. Chemotherapy-induced cell death in primary cerebellar granule neurons but not in astrocytes: in vitro paradigm of differential neurotoxicity. *J. Neurochem*. **91**, 1067–1074 (2004). 15569250 10.1111/j.1471-4159.2004.02774.x

[5] Cepeda, V. et al. Biochemical mechanisms of cisplatin cytotoxicity. *Anti-Cancer Agents Med. Chem. (Form. Curr. Med. Chem. Anti-Cancer Agents)* **7**, 3–18 (2007). 10.2174/187152007779314044 17266502

[6] Manohar, S. & Leung, N. Cisplatin nephrotoxicity: a review of the literature. *J. Nephrol.* **31**, 15–25 (2018). 28382507 10.1007/s40620-017-0392-z

[7] Yadav, Y. C. Effect of cisplatin on pancreas and testes in Wistar rats: biochemical parameters and histology. *Heliyon* **5**, (2019). 10.1016/j.heliyon.2019.e02247 PMC6700420 31453403

[8] Comeau, T. B., Epstein, J. B. & Migas, C. Taste and smell dysfunction in patients receiving chemotherapy: a review of current knowledge. *Support. Care Cancer*. **9**, 575–580 (2001). 11762967 10.1007/s005200100279

[9] Perše, M. Cisplatin mouse models: treatment, toxicity and translatability. *Biomedicines* **9**, 1406 (2021). 34680523 10.3390/biomedicines9101406 PMC8533586

[10] Khalaf, A. A., Ahmed, W. M. S., Moselhy, W. A., Abdel-Halim, B. R. & Ibrahim, M. A. Protective effects of selenium and nano-selenium on bisphenol-induced reproductive toxicity in male rats. *Hum. Exp. Toxicol.* **38**, 398–408 (2019). 30526071 10.1177/0960327118816134

[11] Al-Duais, M. A. et al. The anticancer activity of fucoidan coated selenium nanoparticles and Curcumin nanoparticles against colorectal cancer lines. *Sci. Rep.* **15**, 287 (2025). 39747357 10.1038/s41598-024-82687-y PMC11697394

[12] Bisht, N., Phalswal, P. & Khanna, P. K. Selenium nanoparticles: A review on synthesis and biomedical applications. *Mater. Adv.* **3**, 1415–1431 (2022).

[13] Alhawiti, A. S. Citric acid-mediated green synthesis of selenium nanoparticles: antioxidant, antimicrobial, and anticoagulant potential applications. *Biomass Convers. Biorefin*. **14**, 6581–6590 (2024). 10.1007/s13399-022-02798-2 PMC9126098 35646508

[14] Ciesielska-Figlon, K., Wojciechowicz, K., Wardowska, A. & Lisowska, K. A. The Immunomodulatory effect of Nigella sativa. *Antioxidants* **12**, 1340 (2023). 37507880 10.3390/antiox12071340 PMC10376245

[15] Said, A. M., Atwa, S. A. E. & Khalifa, O. A. Ameliorating effect of gum Arabic and Lemongrass on chronic kidney disease induced experimentally in rats. *Bull. Natl. Res. Cent.* **43**, 1–8 (2019).

[16] Ali, B. H., Ziada, A. & Blunden, G. Biological effects of gum arabic: a review of some recent research. *Food Chem. Toxicol.* **47**, 1–8 (2009). 18672018 10.1016/j.fct.2008.07.001

[17] Elshama, S. S., El-Kenawy, A. E., Osman, H. E. H. & Youseef, H. M. Amelioration of indomethacin systemic toxicity by gum Arabic administration in adult albino rats. *Int. J. Med. Plants Altern. Med.* **2**, 32–46 (2014).

[18] Mohammed, A. & Mohammed, E. Estimation of the active components in gum Arabic collected from Western Sudan. *Int. J. Sci. Res.* **6**, 2319–7064 (2015).

[19] Pyrzynska, K. & Sentkowska, A. Biosynthesis of selenium nanoparticles using plant extracts. *J. Nanostructure Chem. Vol*. **12**, 467–480 (2022).

[20] Hanafy, N. & Farid, N. Protective effect of Nano-Moringa Oleifera leaves extract and/or low doses of γ-Irradiation on acute pancreatitis model induced in rats. *Int. J. Theoretical Appl. Res.* **2**, 215–224 (2023).

[21] Wang, X. et al. Development and characterization of zein/gum Arabic nanocomposites incorporated edible films for improving strawberry preservation. *Adv. Compos. Hybrid. Mater.* **7**, 249 (2024).

[22] Montes Ruiz-Cabello, F. J., Trefalt, G., Maroni, P. & Borkovec, M. Electric double-layer potentials and surface regulation properties measured by colloidal-probe atomic force microscopy. *Phys. Rev. E*. **90**, 012301 (2014). 10.1103/PhysRevE.90.012301 25122297

[23] Hassanin, K. M. A., Abd El-Kawi, S. H. & Hashem, K. The prospective protective effect of selenium nanoparticles against chromium-induced oxidative and cellular damage in rat thyroid. *Int. J. Nanomed.* **8**, 1713–1720 (2013). 10.2147/IJN.S42736 PMC3646488 23658489

[24] Gado, A. M. & Aldahmash, B. A. Antioxidant effect of Arabic gum against mercuric chloride-induced nephrotoxicity. *Drug Des. Devel Ther.* **7**, 1245–1252 (2013). 24174869 10.2147/DDDT.S50928 PMC3808154

[25] Qutifan, S. et al. Melatonin mitigates cisplatin-induced cognitive impairment in rats and improves hippocampal dendritic spine density. *Neuroreport* **35**, 657–663 (2024). 38813907 10.1097/WNR.0000000000002049

[26] Hua, Z., Gibson, S. L., Foster, T. H. & Hilf, R. Effectiveness of δ-aminolevulinic acid-induced protoporphyrin as a photosensitizer for photodynamic therapy in vivo. *Cancer Res.* **55**, 1723–1731 (1995). 7712481

[27] Abo-Ouf, M. Effect of Fluoxetine hydrochloride on the histological structure of the cerebellar cortex of albino rat offspring of treated mothers. *Al-Azhar Med. J.* **47** (3), 603–634 (2018).

[28] Omar, A. A., AH, Gad, M. F., Refaie, A. A., Abdelhafez, H. M. & Mossa, A. T. H. *Benchmark Dose Approach to DNA and Liver Damage by Chlorpyrifos and Imidacloprid in Male Rats: The Protective Effect of a Clove-Oil-Based Nanoemulsion Loaded with Pomegranate Peel Extract. Toxics*, 11 569. (2023). 10.3390/toxics11070569 PMC10383980 37505536

[29] Gad, E. S. et al. Cilostazol counteracts mitochondrial dysfunction in hepatic encephalopathy rat model: insights into the role of cAMP. *Eur. J. Pharmacol.* **987**, 177–194 (2025). /AMPK/SIRT1/PINK-1/parkin hub and p-CREB/BDNF/TrkB neuroprotective trajectory. 10.1016/j.ejphar.2024.177194 39667427

[30] Kandeal, H. A., Eid, F. A., Abdelhafez, H. & Abd El-Hady, A. M. Role of acacia Arabica gum in reducing the impair alterations in liver tissue of irradiated albino rats-Histopathological study. *Int. J. Theoretical Appl. Res.* **1**, 18–26 (2022).

[31] Elsharkawy, L. K., Barghash, S. M., El-Nour, B. M. A., Labib, W. & Sadek, A. S. M. Infection survey, molecular, pathogenicity, and morphological characteristics of sarcocystis species naturally infected water buffaloes (Bubalus bubalis) in Egypt. *BMC Vet. Res.* **20**, 578 (2024). 39716208 10.1186/s12917-024-04408-x PMC11664923

[32] Wishart, D. S. et al. DrugBank 5.0: a major update to the drugbank database for 2018. *Nucleic Acids Res.* **46**, 1074–1082 (2018). 10.1093/nar/gkx1037 PMC5753335 29126136

[33] Hähnke, V. D., Kim, S. & Bolton, E. E. PubChem chemical structure standardization. *J. Cheminform*. **10**, 36 (2018). 30097821 10.1186/s13321-018-0293-8 PMC6086778

[34] Pawar, S. S. & Rohane, S. H. Review on discovery studio: an important tool for molecular Docking. *Asian J. Res. Chem.* **14**, 1–3 (2021).

[35] Daina, A., Michielin, O. & Zoete, V. SwissADME: a free web tool to evaluate pharmacokinetics, drug-likeness and medicinal chemistry friendliness of small molecules. *Sci. Rep.* 7. (2017). 10.1038/srep42717 PMC5335600 28256516

[36] Jiang, H. et al. Biological activities and solubilization methodologies of naringin. *Foods* **12**, 2327 (2023). 37372538 10.3390/foods12122327 PMC10297695

[37] Elwan, A. G., Mohamed, T. M., Beltagy, D. M. & El Gamal, D. M. The therapeutic role of naringenin nanoparticles on hepatocellular carcinoma. *BMC Pharmacol. Toxicol.* **26**, 3 (2025). 39754228 10.1186/s40360-024-00823-w PMC11697747

[38] Bai, Y. et al. Pharmacokinetics and metabolism of naringin and active metabolite naringenin in rats, dogs, humans, and the differences between species. *Front. Pharmacol.* **11**, 364 (2020). 32292344 10.3389/fphar.2020.00364 PMC7118210

[39] Batiha, G. E. S. et al. Elewa Y H A. The Pharmacological activity, biochemical properties, and pharmacokinetics of the major natural polyphenolic flavonoid: Quercetin. *Foods* **9**, 374 (2020). 32210182 10.3390/foods9030374 PMC7143931

[40] Parveen, S., Bhat, I. U. H. & Bhat, R. Kaempferol and its derivatives: biological activities and therapeutic potential. *Asian Pac. J. Trop. Biomed.* **13**, 411–420 (2023).

[41] Amjad, E., Sokouti, B. & Asnaashari, S. A systematic review of anti-cancer roles and mechanisms of Kaempferol as a natural compound. *Cancer Cell. Int.* **22**, 260 (2022). 35986346 10.1186/s12935-022-02673-0 PMC9392350

[42] Taban, K., İlhan, M., Süntar, I. & Luteolin Advances on Resources, Biosynthesis Pathway, Bioavailability, Bioactivity, and Pharmacology. In: *Handbook of Dietary Flavonoids.* 1–37. (2023).

[43] Bae, J., Kim, N., Shin, Y., Kim, S. Y. & Kim, Y. J. Activity of catechins and their applications. *Biomedical Dermatology*. **4**, 1–10 (2020).

[44] Tabassum, N., Varras, P. C., Arshad, F., Choudhary, M. I. & Yousuf, S. Biological activity tuning of antibacterial Urotropine via co-crystallization: synthesis, biological activity evaluation and computational insight. *CrystEngComm* **22**, 349–3450 (2020).

[45] Zaman, A. et al. Exploring Pharmacological potentials of p-coumaric acid: a prospective phytochemical for drug discovery. *Bangladesh Pharm. J.* **26**, 185–194 (2023).

[46] Aldaba-Muruato, L. R. et al. Therapeutic perspectives of p-coumaric acid: Anti-necrotic, anti-cholestatic and anti-amoebic activities. *World Acad. Sci. J.* **3**, 47 (2021).

[47] Ruwizhi, N. & Aderibigbe, B. A. Cinnamic acid derivatives and their biological efficacy. *Int. J. Mol. Sci.* **21**, 57–12 (2020). 10.3390/ijms21165712 PMC7460980 32784935

[48] Aijaz, M. et al. Chemical, biological, and Pharmacological prospects of caffeic acid. *Biointerface Res. Appl. Chem.* **13**, 324 (2022).

[49] Silva, H. & Lopes, N. M. F. Cardiovascular effects of caffeic acid and its derivatives: a comprehensive review. *Front. Physiol.* **11**, 595–516 (2020). 33343392 10.3389/fphys.2020.595516 PMC7739266

[50] Harwansh, R. K. et al. Recent advancements in Gallic Acid-Based drug delivery: Applications, clinical Trials, and future directions. *Pharmaceutics* **16**, 1202 (2024). 39339238 10.3390/pharmaceutics16091202 PMC11435332

[51] Pyrzynska, K. Ferulic acid a brief review of its extraction, bioavailability and biological activity. *Separations* **11**, 1204 (2024).

[52] Khan, K. A. et al. Ferulic acid: therapeutic potential due to its antioxidant properties, role in plant growth, and stress tolerance. *Plant. Growth Regul. *1–25. (2024).

[53] Tang, F. et al. Research on the Mechanism of Kaempferol for Treating Senile Osteoporosis by Network Pharmacology and Molecular Docking*. Evidence-based Complementary and Alternative Medicine.* (2022). 10.1155/2022/6741995 PMC8831051 35154351

[54] Zhong, W. et al. Network Pharmacology and molecular docking-based investigation on traditional Chinese medicine astragalus Membranaceus in oral ulcer treatment. *Med. (United States)*. **102**, E34744 (2023). 10.1097/MD.0000000000034744 PMC10470703 37653793

[55] Jiao, X. et al. Study on the mechanism of prunella vulgaris L on diabetes mellitus complicated with hypertension based on network Pharmacology and molecular Docking analyses. *J. Diabetes Res.* (2021). 10.1155/2021/9949302 PMC8536441 34692849

[56] Huang, X. Z. et al. *Traditional Chinese medicine drynariae rhizoma and cuscuta chinensis suppress osteoarthritis by Quercetin-AKT1 and Luteolin-IL6/VEGFA direct binding*. 10.21203/rs.3.rs-409882/v1 (2021).

[57] Wang, T. et al. Exploring the mechanism of Luteolin by regulating microglia polarization based on network Pharmacology and in vitro experiments. *Sci. Rep. *13. (2023). 10.1038/s41598-023-41101-9 PMC10447507 37612462

[58] Megantara, S., Mutakin, M., Halimah, E., Febrina, E. & Levita, J. Molecular interaction of the downstream executioner cysteine aspartyl proteases (Caspase-3 and caspase-7) with corilagin, quercetin, rutin, kaempferol, Gallic acid, and Geraniin of acalypha Wilkesiana müll.arg. *Rasayan J. Chem.* **13**, 1321–1329 (2020).

[59] Tao, S. Y. et al. Network pharmacology-based strategy combined with molecular Docking to explore the potential mechanism of Agarwood against recurrent aphthous stomatitis. *Med. (United States)*. **103**, E37660 (2024). 10.1097/MD.0000000000037660 PMC10977553 38552047

[60] Chen, Y. et al. Drug repurposing based on the similarity gene expression signatures to explore for potential indications of quercetin: a case study of multiple sclerosis. *Front. Chem.* **11**, (2023). 10.3389/fchem.2023.1250043 PMC10514366 37744058

[61] Oz, M., Atalik, K. E. N., Yerlikaya, F. H. & Demir, E. A. Curcumin alleviates cisplatin-induced learning and memory impairments. *Neurobiol. Learn. Mem.* **123**, 143–149 (2015). 10.1016/j.nlm.2015.05.001 25982942

[62] Abou-El-Naga, A. M. et al. Restorative effects of momordica Charantia extract on cerebellar GFAP and NGF expression in pregnant diabetic rats and their offspring. *PLoS One*. **20**, e0321022 (2025). 40184394 10.1371/journal.pone.0321022 PMC11970674

[63] Elnahas Sh, M., Mansour, H. A., El-Sawi, M. R. & Abou-El-Naga, A. M. Therapeutic effect of momordica Charantia on cardiomyopathy in a diabetic maternal rat model. *J. Experimental Zool. Part. A: Ecol. Integr. Physiol.*10.1002/jez.2854 (2024). 10.1002/jez.2854 38973290

[64] Prasad, N., Thombare, N., Sharma, S. C. & Kumar, S. Gum arabic–A versatile natural gum: A review on production, processing, properties and applications. *Ind. Crops Prod.* **187**, 115–304 (2022).

[65] Rajeshkumar, S., Ganesh, L. & Santhoshkumar, J. Selenium nanoparticles as therapeutic agents in neurodegenerative diseases. *Nanobiotechnol. Neurodegenerative Dis. *209–224. (2019).

[66] Gholamigeravand, B. et al. Administration of selenium nanoparticles reverses streptozotocin-induced neurotoxicity in the male rats. *Metab. Brain Dis.* **36**, 1259–1266 (2021). 33826055 10.1007/s11011-021-00713-8

[67] Hamdani, A. M., Wani, I. A., Bhat, N. A. & Masoodi, F. A. Chemical composition, total phenolic content, antioxidant and antinutritional characterisation of exudate gums. *Food Biosci.* **23**, 67–74 (2018).

[68] Kaurinovic, B. & Vastag, D. Flavonoids and phenolic acids as potential natural antioxidants. *Antioxidants* **2**, 1–14 (2019).

[69] Sies, H., Berndt, C. & Jones, D. P. Oxidative stress. *Annu. Rev. Biochem.* **86**, 715–748 (2017). 28441057 10.1146/annurev-biochem-061516-045037

[70] Kim, G. H., Kim, J. E., Rhie, S. J. & Yoon, S. The role of oxidative stress in neurodegenerative diseases. *Exp. Neurobiol.* **24**, 325–340 (2015). 26713080 10.5607/en.2015.24.4.325 PMC4688332

[71] Kong HuiLing, K. H. et al. Synthesis and antioxidant properties of gum arabic-stabilized selenium nanoparticles. *Int. J. Biol. Macromol.* **65**, 155–162 (2014). 24418338 10.1016/j.ijbiomac.2014.01.011

[72] Hasanzadeh, M. et al. Comparative study of biosynthesizing selenium nanoparticles by gum Arabic and Poly anionic cellulose to prevent Radiation-Induced death in Chinese hamster ovary (CHO) cells. *Front. Biomedical Technol.*10.18502/fbt.v11i2.15340 (2024).

[73] Ashraf, H. et al. Latent potential of multifunctional selenium nanoparticles in neurological diseases and altered gut microbiota. *Materials* **16**, 699 (2023). 36676436 10.3390/ma16020699 PMC9862321

[74] Arslan, B. et al. Investigation of the effects of Epigallocatechin-3-Gallate on Caspase-3, IL-1α, IL-6, P53 and HO-1 gene expressions against Cisplatin-Induced pancreatic tissue injury in rats. *Pharm. Chem. J.* **58**, 974–983 (2024).

[75] Balaraju, P. C. et al. Exploring the therapeutic potential of selenium nanoparticles in central nervous system disorders: A nanomedicine approach. *Int. J. Pharm. Investig* **14**, (2024).

[76] Sahoo, G., Samal, D., Khandayataray, P. & Murthy, M. K. A review on caspases: key regulators of biological activities and apoptosis. *Mol. Neurobiol.* **60**, 5805–5837 (2023). 37349620 10.1007/s12035-023-03433-5

[77] Huo, Y., Zong, Z., Wang, Q., Zhang, Z. & Deng, H. ISG15 Silencing increases cisplatin resistance via activating p53-mediated cell DNA repair. *Oncotarget* **8**, 107452–107461 (2017). 29296177 10.18632/oncotarget.22488 PMC5746079

[78] Mantovani, F., Collavin, L. & Del Sal, G. Mutant p53 as a guardian of the cancer cell. *Cell. Death Differ.* **26**, 199–212 (2019). 30538286 10.1038/s41418-018-0246-9 PMC6329812

[79] Wongtawatchai, T., Agthong, S., Kaewsema, A. & Chentanez, K. Sex-related differences in cisplatin-induced neuropathy in rats. *J. Med. Assoc. Thai.* **92**, 1485 (2011). 19938741

[80] Mohamed, D. S., Shehata, O., Labib, M. M. & Shaban, N. S. Integrated in vivo and in Silico evaluation of sweet Basil oil as a protective agent against cisplatin-induced neurotoxicity in mice. *Beni Suef Univ. J. Basic. Appl. Sci.* **12**, 65 (2023).

[81] Obaid, S. S. The medical uses of gum Acacia-Gum Arabic (GA) in human. *Acad. J. Res. Sci. Publishing* **1**, (2020).

[82] Elfakharany, S. A. et al. Neuroprotective role of selenium nanoparticles against behavioral, neurobiochemical and histological alterations in rats subjected to chronic restraint stress. *Mol. Neurobiol.* **61**, 10159–10181 (2024). 38703343 10.1007/s12035-024-04196-3 PMC11584447

[83] Attia, A., Matta, C., El Mazoudy, R. & Khalifa, H. Cisplatin-induced neurotoxicity in cerebellar cortex of male mice involves oxidative stress and histopathology. *J. Basic. Appl. Zool.* **82**, 23 (2021).

[84] Alotaibi, M. et al. Alleviation of cisplatin-induced neuropathic pain, neuronal apoptosis, and systemic inflammation in mice by Rapamycin. *Front. Aging Neurosci.* **14**, (2022). 10.3389/fnagi.2022.891593 PMC9554141 36248001

[85] Vezzani, A., Balosso, S. & Ravizza, T. Neuroinflammatory pathways as treatment targets and biomarkers in epilepsy. *Nat. Rev. Neurol.* **15**, 459–472 (2019). 31263255 10.1038/s41582-019-0217-x

[86] Al-Jubori, Y. et al. The efficacy of gum arabic in managing diseases: a systematic review of evidence-based clinical trials. *Biomolecules* **13**, 138 (2023). 36671523 10.3390/biom13010138 PMC9855968

[87] Mokhtar, H. E. L., Hulail, M. A. E., Mahmoud, S. M. & Yousef, D. M. Impact of cisplatin administration on cerebellar cortical structure and locomotor activity of infantile and juvenile albino rats: the role of oxidative stress. *Anat. Sci. Int.* **97**, 30–47 (2022). 34386931 10.1007/s12565-021-00624-9

[88] Abdel Mohsen, A., Ahmed, N., Altaib, Z. & Zaher, S. Effect of cisplatin on cerebellar cortex of albino rat and possible protective role of granulocyte colony stimulating factor versus citrullus lanatus juice: A histological study. *Egypt. J. Histol.* **0**, 0–0 (2019).

[89] O’Callaghan, J. P. & Sriram, K. Glial fibrillary acidic protein and related glial proteins as biomarkers of neurotoxicity. *Expert Opin. Drug Saf.* **4**, 433–442 (2005). 15934851 10.1517/14740338.4.3.433

[90] Eng, L. F. & Ghirnikar, R. S. *GFAP Astrogliosis Brain Pathol.*, ; **4**: 229–237. (1994). 7952264 10.1111/j.1750-3639.1994.tb00838.x

[91] Babiker, R., Kaddam, L. & Mariod, A. The role of gum Arabic as an anti-inflammatory, antioxidant, and immune modulator in the development of COVID-19: A review. *Funct. Food Sci.* **2**, 242 (2022).

[92] Malhotra, S., Welling, M. N., Mantri, S. B. & Desai, K. In vitro and in vivo antioxidant, cytotoxic, and antichronic inflammatory arthritic effect of selenium nanoparticles. *J. Biomed. Mater. Res. B Appl. Biomater.* **104**, 993–1003 (2016). 25994972 10.1002/jbm.b.33448

[93] Meregalli, C. et al. Neurofilament light chain: a specific serum biomarker of axonal damage severity in rat models of Chemotherapy-Induced peripheral neurotoxicity. *Arch. Toxicol.* **94**, 2517–2522 (2020). 32333051 10.1007/s00204-020-02755-w

[94] Varhaug, K. N., Torkildsen, Ø., Myhr, K. M. & Vedeler, C. A. Neurofilament light chain as a biomarker in multiple sclerosis. *Front. Neurol.* 10 (2019). 10.3389/fneur.2019.00338 PMC6460359 31024432

[95] Coppens, S., Lehmann, S., Hopley, C. & Hirtz, C. Neurofilament-light, a promising biomarker: analytical, metrological and clinical challenges. *Int. J. Mol. Sci.* **24**, 11624 (2023). 37511382 10.3390/ijms241411624 PMC10380627

[96] Abdel Mohsen, A. F., Ahmed, N. A. W., Altaib, Z. M. & Zaher, S. M. Effect of cisplatin on cerebellar cortex of albino rat and possible protective role of granulocyte colony stimulating factor versus citrullus lanatus juice: a histological study. *Egypt. J. Histol.* **43**, 702–717 (2020).

[97] Bobylev, I., Joshi, A. R., Barham, M., Neiss, W. F. & Lehmann, H. C. Depletion of Mitofusin-2 causes mitochondrial damage in Cisplatin-Induced neuropathy. *Mol. Neurobiol.* **55**, 1227–1235 (2018). 28110471 10.1007/s12035-016-0364-7

[98] Podratz, J. L. et al. Neurotoxicity to DRG neurons varies between rodent strains treated with cisplatin and bortezomib. *J. Neurol. Sci.* **362**, 131–135 (2016). 26944133 10.1016/j.jns.2015.12.038 PMC4779499

